# Using the avian mutant *talpid^2^* as a disease model for understanding the oral-facial phenotypes of oral-facial-digital syndrome

**DOI:** 10.1242/dmm.020222

**Published:** 2015-08-01

**Authors:** Elizabeth N. Schock, Ching-Fang Chang, Jaime N. Struve, Ya-Ting Chang, Julie Chang, Mary E. Delany, Samantha A. Brugmann

**Affiliations:** 1 Division of Plastic Surgery, Department of Surgery, Cincinnati Children's Hospital Medical Center, Cincinnati, OH 45229, USA; 2 Division of Developmental Biology, Department of Pediatrics, Cincinnati Children's Hospital Medical Center, Cincinnati, OH 45229, USA; 3 University of Cincinnati, Cincinnati, OH 45229, USA; 4 College of Agricultural and Environmental Sciences, Department of Animal Science, University of California Davis, Davis, CA 95616, USA

**Keywords:** Primary cilia, Craniofacial, Neural crest, *talpid^2^*, Ciliopathies, Chicken, Oral-facial-digital syndrome

## Abstract

Oral-facial-digital syndrome (OFD) is a ciliopathy that is characterized by oral-facial abnormalities, including cleft lip and/or palate, broad nasal root, dental anomalies, micrognathia and glossal defects. In addition, these individuals have several other characteristic abnormalities that are typical of a ciliopathy, including polysyndactyly, polycystic kidneys and hypoplasia of the cerebellum. Recently, a subset of OFD cases in humans has been linked to mutations in the centriolar protein C2 Ca^2+^-dependent domain-containing 3 (C2CD3). Our previous work identified mutations in *C2CD3* as the causal genetic lesion for the avian *talpid^2^* mutant. Based on this common genetic etiology, we re-examined the *talpid^2^* mutant biochemically and phenotypically for characteristics of OFD. We found that, as in OFD-affected individuals, protein-protein interactions between C2CD3 and oral-facial-digital syndrome 1 protein (OFD1) are reduced in *talpid^2^* cells. Furthermore, we found that all common phenotypes were conserved between OFD-affected individuals and avian *talpid^2^* mutants. In light of these findings, we utilized the *talpid^2^* model to examine the cellular basis for the oral-facial phenotypes present in OFD. Specifically, we examined the development and differentiation of cranial neural crest cells (CNCCs) when C2CD3-dependent ciliogenesis was impaired. Our studies suggest that although disruptions of C2CD3-dependent ciliogenesis do not affect CNCC specification or proliferation, CNCC migration and differentiation are disrupted. Loss of C2CD3-dependent ciliogenesis affects the dispersion and directional persistence of migratory CNCCs. Furthermore, loss of C2CD3-dependent ciliogenesis results in dysmorphic and enlarged CNCC-derived facial cartilages. Thus, these findings suggest that aberrant CNCC migration and differentiation could contribute to the pathology of oral-facial defects in OFD.

## Abstract (precis)

**Summary:** This study utilizes a naturally occurring avian mutant known as *talpid^2^* to determine the cellular basis for the oral-facial phenotypes present in oral-facial-digital syndrome.

## INTRODUCTION

Primary cilia are non-motile, microtubule-based organelles that function as cellular antennas to coordinate the transduction of several signaling pathways. Ciliopathies are a growing class of disease, the cellular etiology of which lies in either disrupted structure or function of the primary cilia. Common clinical features of ciliopathies are broad and include renal cystic disease, polydactyly, situs inversus, retinitis pigmentosa, hepatic disease and mental retardation (Baker and Beales, 2009). Of the known human ciliopathies, approximately 30% are primarily defined by their oral-facial phenotype (Zaghloul and Brugmann, 2011). The most well-known examples of oral-facial ciliopathies are Bardet-Biedl syndrome, Joubert syndrome, Meckel-Gruber syndrome and oral-facial-digital syndrome (OFD) (Beales et al., 1999; Ferrante et al., 2001; Fraser and Lytwyn, 1981; Lorda-Sanchez et al., 2001; Maria et al., 1999). For many of these syndromes, both the genetic basis and the cellular processes that are affected remain unclear.

Cranial neural crest cells (CNCCs) make a large contribution to the developing oral-facial complex. The facial skeleton, melanocytes, glia and smooth muscle are directly derived from CNCCs (Le Douarin et al., 2004), whereas other oral-facial structures, such as teeth and tongue, only require a partial contribution from the CNCCs (Chai et al., 2000). Before CNCCs can differentiate into their final lineage, they undergo several cellular processes. CNCCs are specified at the dorsal neural tube, and migrate ventrolaterally into the developing facial primordia and brachial arches. CNCCs must then proliferate to give the developing facial prominences their mass and shape. Later, CNCCs differentiate into various cell types, including those that make up the skeletal elements of the face (Noden and Trainor, 2005). Given the considerable oral-facial phenotypes present in many ciliopathies, we wanted to explore the role of primary cilia during CNCC development.

*talpid^2^* (*ta^2^*) is a naturally occurring avian mutant that is best characterized by severe polydactyly and its oral-facial phenotype (Abbott et al., 1959, 1960; Dvorak and Fallon, 1991; Schneider et al., 1999). The faces of affected *ta^2^* embryos are characterized by dysmorphic frontonasal prominences, facial clefting, hypoplastic maxillary prominences, incomplete fusion of the primary palate and hypoglossia (Chang et al., 2014). The *ta^2^* mutant itself was first identified in the 1960s; however, the cellular and genetic basis for this mutant remained unknown for decades. Our recent work has determined that the *ta^2^* mutation affects ciliogenesis through a deletion mutation in *C2CD3* (Brugmann et al., 2010; Chang et al., 2014), a centriolar protein that is required for ciliogenesis (Hoover et al., 2008). Concurrently, mutations in *C2CD3* were found in a subset of individuals with OFD (Thauvin-Robinet et al., 2014). Herein, we utilize the avian *ta^2^* mutant to determine the cellular etiology of the common oral-facial phenotypes present in OFD-affected individuals. Specifically, we examine how C2CD3-dependent ciliogenesis affects the development of CNCCs. Taken together, these experiments elucidate the cellular mechanism by which ciliary dysfunction leads to oral-facial anomalies in OFD.

**TRANSLATIONAL IMPACT**

**Clinical issue**

Ciliopathies are a class of diseases caused by defects in primary cilia, organelles that coordinate the transduction of several cellular signaling pathways. Individuals with ciliopathies present with a wide range of phenotypes that often involve multiple organ systems. Oral-facial-digital syndrome (OFD) is a ciliopathy that is primarily characterized by severe oral-facial and digit defects, such as polysyndactyly (extra or fused fingers and toes). To date, only two genes have been identified as being solely causative of OFD – *OFD1* and *C2CD3*. Both genes give rise to proteins that are important for ciliogenesis (the formation of cilia). Individuals with OFD are treated for their symptoms and undergo multiple surgeries to correct malformations. They might also require speech therapy and special education. Although OFD can be diagnosed *in utero*, the cellular and molecular basis for this syndrome is unclear. Animal models are essential to provide valuable insights into the etiology of the disease phenotype in OFD.

**Results**

Here, the authors use a naturally occurring avian mutant known as *talpid^2^* to determine the cellular basis for the oral-facial phenotypes present in OFD. Similar to a subset of individuals with OFD, *talpid^2^* mutants have mutations in the ciliary gene *C2CD3*. *talpid^2^* mutants display strikingly similar phenotypes to individuals with OFD, including cleft lip and/or palate, ectopic teeth, glossal (tongue) defects, polydactyly, polycystic kidneys and brain defects. To better understand the cellular etiology for the oral-facial defects in OFD-affected individuals and *talpid*^2^ mutants, the authors examine a population of embryonic cells called cranial neural crest cells (CNCCs), which give rise to a large portion of the oral-facial complex that is affected in OFD. Their results indicate that CNCC specification and proliferation are unaltered in *talpid^2^* mutants, but that the migration and differentiation of these cells are aberrant. Specifically, loss of C2CD3-dependent ciliogenesis affects the dispersion and directional persistence of migratory CNCCs, and results in mis-shapen and enlarged CNCC-derived facial cartilage.

**Implications and future directions**

These findings suggest that the oral-facial defects in individuals with OFD might be due to disruptions in CNCC migration and differentiation during development, thereby highlighting two cellular processes that might be crucial for the onset of this disease. Understanding when and how potential therapeutic agents could have an impact on OFD is an essential step toward more effective treatment options for affected individuals. Thus, future work will focus on understanding the mechanism that is responsible for altered migration and differentiation in CNCCs that lack C2CD3-dependent ciliogenesis.

## RESULTS

### *talpid^2^* is an avian model for human OFD

*ta^2^* is a naturally occurring avian mutation, and homozygous embryos (*ta^2^/ta^2^*) exhibit a characteristic phenotype, including oral-facial and limb defects (Abbott et al., 1959, 1960; Chang et al., 2014; Harris et al., 2006; Schneider et al., 1999). Our previous work has identified *ta^2^* as a ciliopathic mutant (Brugmann et al., 2010; Chang et al., 2014). Specifically, we identified the causal genetic lesion as a 19-bp deletion at the 3′-end of *C2CD3* (Chang et al., 2014), a gene that is important for centriole elongation and primary cilia formation (Hoover et al., 2008; Thauvin-Robinet et al., 2014; Ye et al., 2014). Recently, mutations in *C2CD3* have also been identified as the causal genetic lesion for a subset of individuals with OFD (Thauvin-Robinet et al., 2014). In light of these genetic findings, we re-examined *ta^2^* embryos both phenotypically and biochemically to determine whether they could be classified as a model for OFD.

**Fig. 1.**
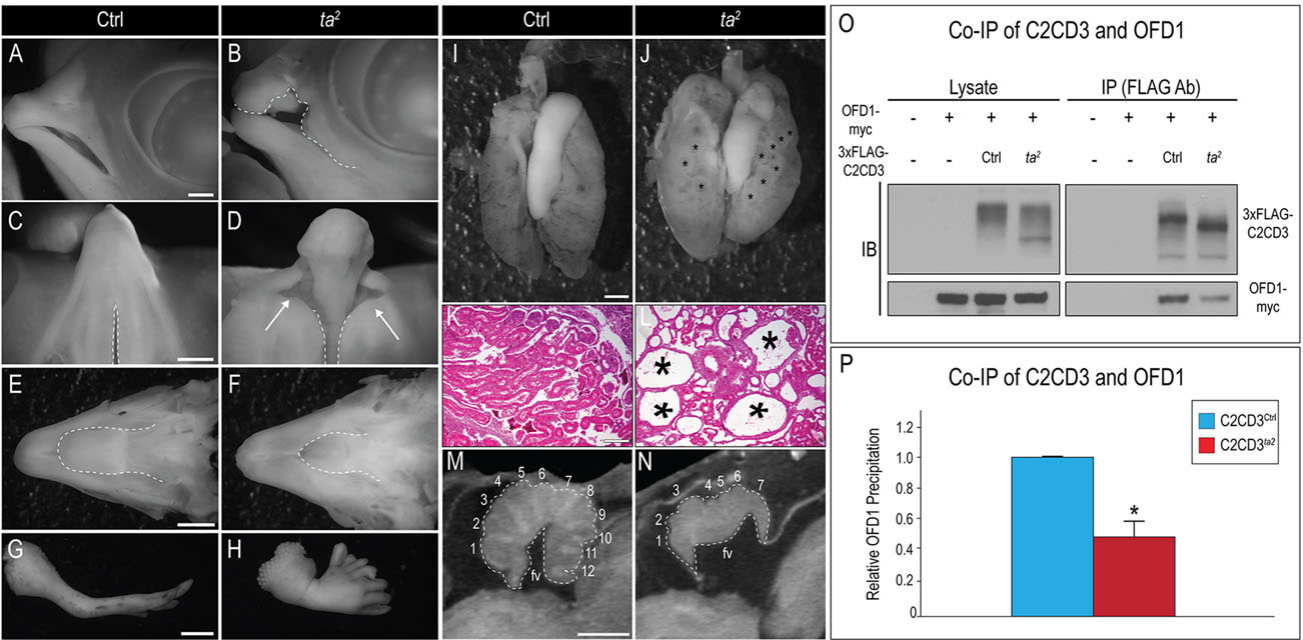
**The avian mutant *ta^2^* phenocopies human OFD.** (A-H) Whole-mount images of day-10 control and *ta^2^* embryos. (A,B) Lateral view of a control (Ctrl, A) and a *ta^2^* face with a cleft lip (B, dotted white line). (C,D) Ventral view of control and a *ta^2^* palate with cleft primary palate (D, white arrows). Compare the width of the naturally occurring cleft of the secondary palate in C and of the pathological cleft in D (dotted white lines). (E,F) Dorsal view of a control and a *ta^2^* mandible. (F) *ta^2^* embryos exhibit hypoglossia (compare dotted white lines in E,F). (G,H) Lateral view of a control and a *ta^2^* forelimb. (H) *ta^2^* embryos exhibit severe polydactyly. (I,J) Whole-mount image of day-13 control and *ta^2^* kidneys. Polycystic kidneys are denoted by black asterisks (J). (K,L) Hematoxylin and eosin staining on day-13 control and *ta^2^* kidneys; cysts are marked with black asterisks (L). (M,N) Micro-CT images of day-13 control and *ta^2^* cerebella with the folia numbered. fv, fourth ventricle. (O,P) Co-immunoprecipitation (Co-IP) of 3×FLAG-C2CD3^Ctrl^ and 3×FLAG-C2CD3*^ta2^* with OFD1-Myc. IB, immunoblot; IP, immunoprecipitation. (P) Quantification of Co-IP. 3×FLAG-C2CD3*^ta2^* has a significantly reduced ability to bind to OFD1-Myc, *P<0.01. Error bars indicate s.e.m. Scale bars: 750 μm (A,B); 1000 μm (C,D); 1150 μm (E,F); 2000 μm (G,H); 700 μm (I,J); 175 μm (K,L); 325 mm (M,N).

Phenotypically, we found a remarkable resemblance between *ta^2^* mutants and OFD-affected individuals. As previously shown, *ta^2^* embryos displayed a myriad of oral-facial defects, including cleft lip and/or palate, ectopic archosaurian-like first generation teeth, hypo- or aglossia (Fig. 1A-F, data not shown) and polydactylous limbs (Fig. 1G,H) (Abbott et al., 1959, 1960; Brugmann et al., 2010; Chang et al., 2014; Harris et al., 2006; Schneider et al., 1999). Additionally, whole-mount and histological examination revealed that *ta^2^* kidneys were polycystic (Fig. 1I-L), consistent with frequently reported kidney defects in OFD (Gorlin et al., 1990). Finally, OFD-affected individuals can present with cerebellar vermal hypoplasia or agenesis, and an enlarged fourth ventricle (Gorlin et al., 1990; Poretti et al., 2008; Thauvin-Robinet et al., 2014). Using micro*-*computed tomography (micro-CT), we analyzed the gross morphology of the avian brain and detected analogous malformations in the cerebellum of *ta^2^* mutants. Specifically, mutants exhibited hypoplasia of the cerebellum with a reduced number of folia, agenesis of the cerebellar vermis and an enlarged fourth ventricle (Fig. 1M,N; control *n*=3, *ta^2^* *n*=3). Taken together, the co-presentation of these symptoms supports our hypothesis that *ta^2^* could be an animal model for OFD.

To further test our hypothesis that *ta^2^* is a bona fide animal model of OFD, we performed biochemical analyses. The majority of OFD cases have been linked to mutations in oral-facial-digital syndrome 1 (OFD1), a distal centriolar protein that restricts centriole elongation (Ferrante et al., 2003, 2001, 2006; Singla et al., 2010). Previous studies have shown that protein-protein interactions between OFD1 and C2CD3 are impaired in OFD cases (Thauvin-Robinet et al., 2014). To test whether the *ta^2^* mutant C2CD3 protein (C2CD3*^ta2^*) was able to physically interact with OFD1, we cloned the avian ortholog of OFD1, transiently transfected constructs for both control OFD1 and control C2CD3 (C2CD3*^Ctrl^*) or C2CD3*^ta2^*, and performed co-immunoprecipitation assays in chicken embryonic fibroblasts (CEFs) (Fig. 1O,P). Avian OFD1 and C2CD3*^Ctrl^* physically interacted in CEFs; by contrast, there was a significant reduction in the amount of C2CD3*^ta2^* co-precipitated with OFD1 (Fig. 1O,P; supplementary material Fig. S1). These results closely mimic those observed in humans. Thus, from genetic, phenotypic and biochemical evaluations, our data strongly suggest that *ta^2^* is a bona fide animal model for human OFD. We next sought to understand the cellular mechanism behind the oral-facial phenotypes of OFD.

### Primary cilia extend from cranial neural crest cells during all ontogenic phases

**Fig. 2.**
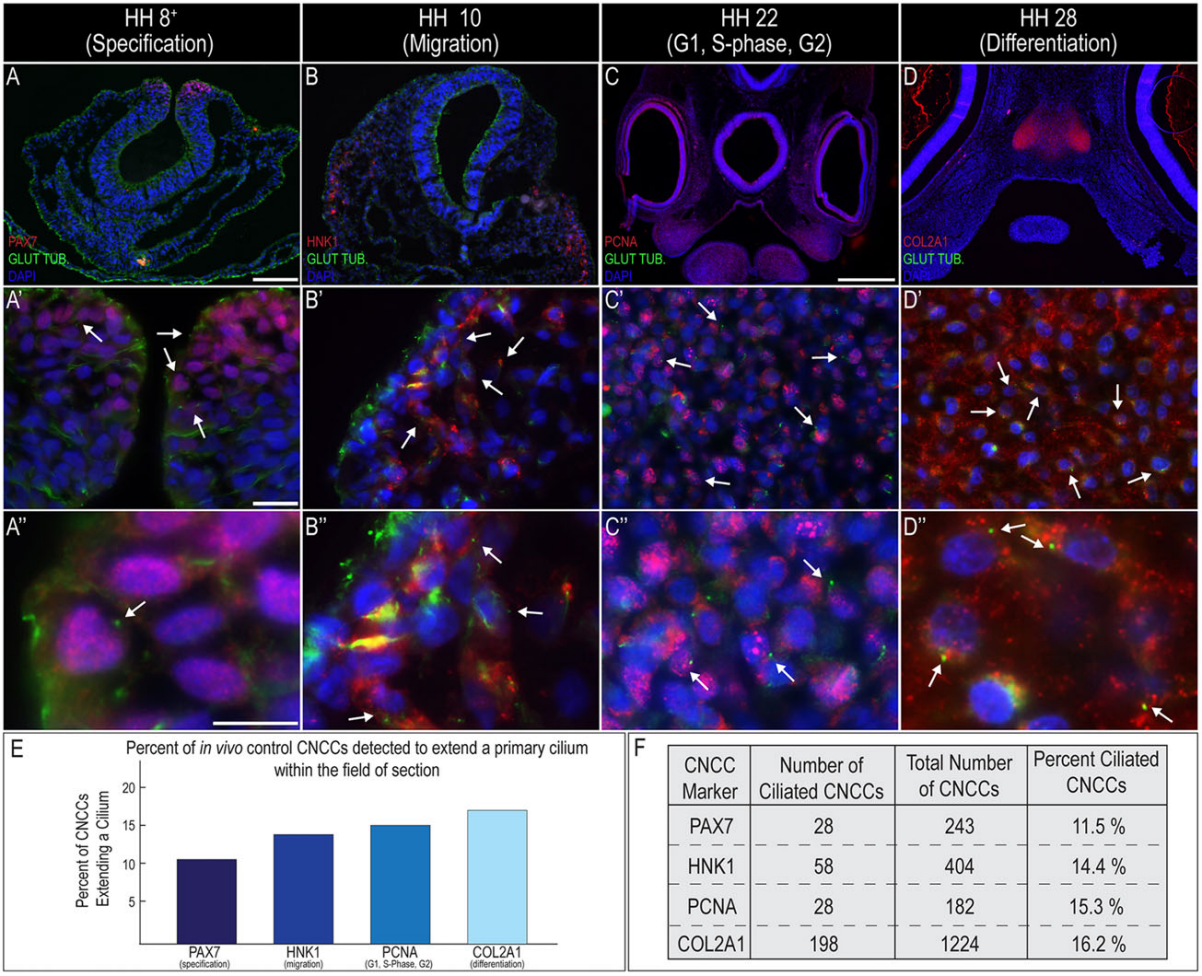
**Primary cilia extend from CNCCs during all ontogenic phases.** (A-D″) Cranial sections of control embryos co-immunostained for glutamylated-tubulin (green) and CNCCs (red) at different ontogenic stages. (A-A″) HH8^+^ (specification; PAX7), (B-B″) HH10 (migration; HNK1), (C-C″) HH22 (G1, S-phase, G2; PCNA) and (D-D″) HH28 (differentiation; COL2A1). Arrows indicate cells positive for both glutamylated-tubulin and CNCC markers. (E,F) Quantification of *in vivo* primary cilia extension on CNCCs. Scale bars: 100 μm (A, applies to B); 1000 μm (C, applies to D); 20 μm (A′, applies to B′-D′); 10 μm (A″, applies to B″-D″).

CNCCs make substantial contributions to the oral-facial complex (Le Douarin et al., 2004; Le Douarin and Dupin, 1993), specifically, the oral-facial regions that are affected in OFD-affected individuals. To determine if and when CNCCs extend primary cilia *in vivo*, we performed co-immunostaining for the ciliary marker glutamylated-tubulin and markers of CNCCs or their derivatives. To determine whether primary cilia were extended during CNCC specification, we performed co-immunostaining for the CNCC marker PAX7 and glutamylated-tubulin. At Hamburger–Hamilton stage (HH) 8^+^, multiple PAX7-positive cells extended a primary cilium (Fig. 2A-A″, arrows). We next examined whether migrating CNCCs extended a primary cilium. At HH10, we detected HNK1-positive cells that extended a primary cilium (Fig. 2B-B″, arrows). Next, we examined the correlation between cell-cycle stages and the presence of a cilium on CNCCs in HH22 embryos. Consistent with other reports, we did not observe any ciliary extension during M-phase, as marked by staining of PHH3 (supplementary material Fig. S2), but we did detect extended cilia during G1, S-phase and G2, as marked by co-staining of glutamylated-tubulin and PCNA (Fig. 2C-C″, arrows). Finally, we examined primary cilia extension on differentiating CNCCs. Although CNCCs can differentiate into a wide variety of cell types, we chose to use cartilage as our readout for differentiation because CNCC-derived cartilage constitutes a substantial portion of the developing face. Co-immunostaining for COL2A1 and glutamylated-tubulin on HH28 embryos showed a robust extension of primary cilia in differentiating chondrocytes (Fig. 2D-D″, arrows). It should be noted that because we performed staining on tissue sections, rather than synchronized cells in culture, we were only able to detect a percentage of the cells that extended primary cilia during these stages (Fig. 2E,F). We have previously isolated and cultured facial mesenchyme under serum-starved conditions and observed the expected 65-75% of cells exhibiting ciliary extension (Chang et al., 2014). Taken together, these data strongly support the notion that CNCCs extend cilia at all developmental time points, except during active mitosis. Thus, we hypothesized that the oral-facial phenotypes present in OFD-affected individuals are due to defects in the extension of cilia by CNCCs. To test this hypothesis, we next examined whether disruption of C2CD3-dependent ciliogenesis could affect CNCC progression through any of these developmental phases.

### CNCC specification is not affected by loss of C2CD3-dependent ciliogenesis

Both OFD-affected individuals and *ta^2^* mutants present with facial clefting. One possible cause of clefting is CNCC insufficiency (Dixon et al., 2006). To determine whether CNCCs are properly specified when C2CD3-dependent ciliogenesis is disrupted, we performed whole-mount *in situ* hybridization for various neural crest specifier genes, including *Sox10*, *Snai2* and *Pax7*. There was no change in the domains of expression between control and *ta^2^* embryos for any of these genes (supplementary material Fig. S3A-H; *Sox10* control *n*=6, *ta^2^ n*=4; *Snai2* control *n*=22, *ta^2^ n*=5; *Pax7* control *n*=16, *ta^2^ n*=6). We sought to confirm this finding by performing immunostaining of PAX7 on cranial cross-sections of HH8^+^ control and *ta^2^* embryos. There was neither a substantial expansion nor a reduction in the domain of PAX7 staining (supplementary material Fig. S3I-J; control *n*=4, *ta^2^ n*=3). Thus, we concluded that C2CD3-dependent ciliary function is not required for the generation or proper specification of CNCCs and, therefore, is not responsible for the oral-facial phenotypes in OFD.

### Defects in C2CD3-dependent ciliogenesis increase dispersion and reduce directional persistence in migrating CNCCs

**Fig. 3.**
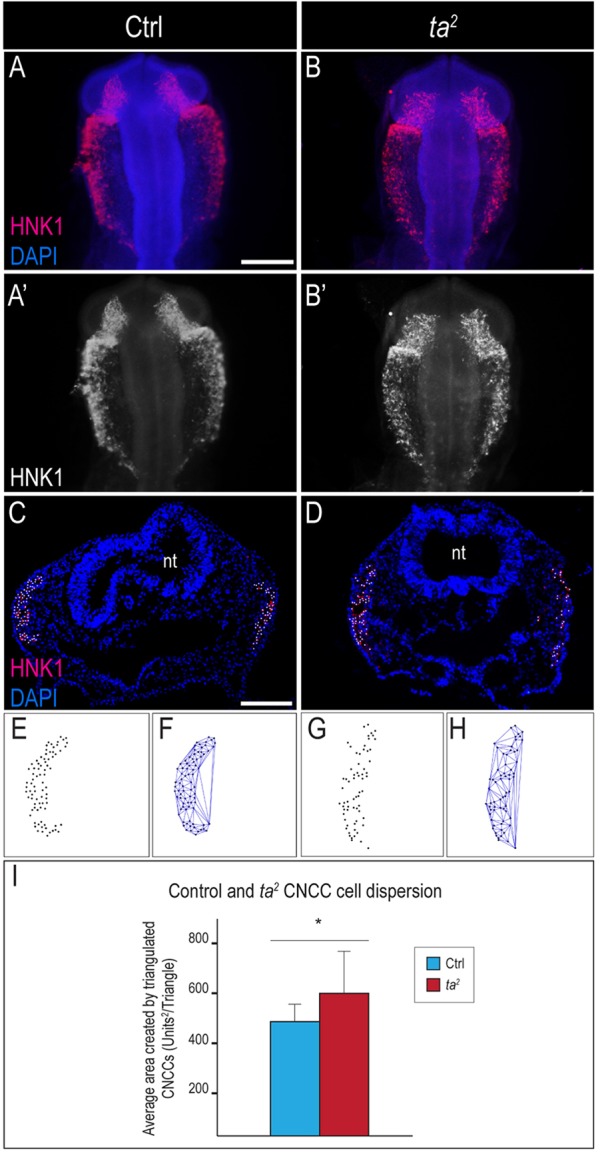
**Loss of C2CD3-dependent ciliogenesis increases CNCC dispersion during migration.** (A-B′) Whole-mount immunostaining of HNK1 on (A,A′) control (Ctrl) and (B,B′) *ta^2^* embryos at HH10. (C,D) HNK1 immunostaining on cranial cross-sections of HH11 control and *ta^2^* embryos. Nuclei of HNK1-positive cells are marked with a white dot. (E-H) CNCC nuclei dispersion and Delaunay triangulation. (E,G) Unilateral representation of migrating CNCC nuclei from embryos in C and D, respectively. (F,H) Delaunay triangulation of migrating CNCC nuclei. *ta^2^* CNCCs showed enhanced dispersion (compare size of triangles in F and H). (I) Quantification of triangulated areas from migrating CNCCs in control and *ta^2^* embryos show that migrating *ta^2^* CNCCs were significantly more disperse, *P<0.05. nt, neural tube. Error bars indicate s.d. Scale bars: 100 μm (A, applies to B,A′,B′); 400 μm (C, applies to D).

For proper facial development, CNCCs must migrate from the dorsal neural tube into the developing facial prominences. Disrupted CNCC migration has previously been associated with facial clefting (He and Soriano, 2013; Vasudevan and Soriano, 2014). For CNCCs to migrate properly into the developing facial prominences, they must collectively move in streams, maintaining close contact with adjacent cells (Kuo and Erickson, 2010; Minoux and Rijli, 2010). Furthermore, it has been shown that these local and transient cell-cell contacts are required for proper CNCC migration (Mayor and Carmona-Fontaine, 2010). To test whether aberrant CNCC migration occurs in cells that lack C2CD3-dependent ciliogenesis, we performed whole-mount immunostaining for HNK1 in HH10 control and *ta^2^* embryos. CNCCs were able to migrate in mutants; however, the results of immunostaining of HNK1 suggested that *ta^2^* CNCCs were more dispersed when compared with control CNCCs (Fig. 3A-B′; control *n*=6, *ta^2^ n*=4).

To confirm and quantify the observation that the loss of C2CD3-dependent ciliogenesis increased the dispersion of migrating CNCCs, we performed Delaunay triangulation – a mathematical application that has been used to determine the density of a set of cells, as previously described (Carmona-Fontaine et al., 2011). The nuclei of migrating CNCCs were marked (Fig. 3C,D; white dots), and images were bisected and imported into ImageJ for analysis (Fig. 3E,G). The Delaunay triangulation algorithm was used to determine the two closest neighbors of a given cell, resulting in the formation of triangles (Fig. 3F,H). The area of the triangles was calculated as a measure of cell dispersion (Fig. 3I; control *n*=12, *ta^2^ n*=10). The average area between triangulated CNCC nuclei was significantly increased in *ta^2^* embryos, suggesting that the loss of C2CD3-dependent ciliogenesis increases the dispersion of migrating CNCCs.

To exclude the possibility that the observed decrease in density was due to fewer cells, increased apoptosis or reduced proliferation of CNCCs, we counted HNK-positive cells, and performed quantitative reverse-transcriptase PCR (qRT-PCR) analysis for *Sox10*, terminal deoxynucleotidyl transferase dUTP nick-end labeling (TUNEL) and immunostaining of PHH3 on both whole-mount and cranial sections of HH10 embryos (supplementary material Fig. S4). Neither the number of migrating CNCCs (supplementary material Fig. S4A; control *n*=11; *ta^2^ n*=10) nor the amount of *Sox10* expression (supplementary material Fig. S4B; control *n*=16, *ta^2^ n*=9) was significantly altered in *ta^2^* mutants. Furthermore, there was no change in the amount of apoptosis (supplementary material Fig. S4C-F; control *n*=13, *ta^2^ n*=6) or proliferation (supplementary material Fig. S4G,H; control *n*=6, *ta^2^ n*=2) of CNCCs between control and *ta^2^* embryos. Given these data, we concluded that the observed increase in dispersion was not a consequence of a reduced number of CNCCs. We next tested whether this increased dispersion correlated with disruption of migratory behavior.

**Fig. 4.**
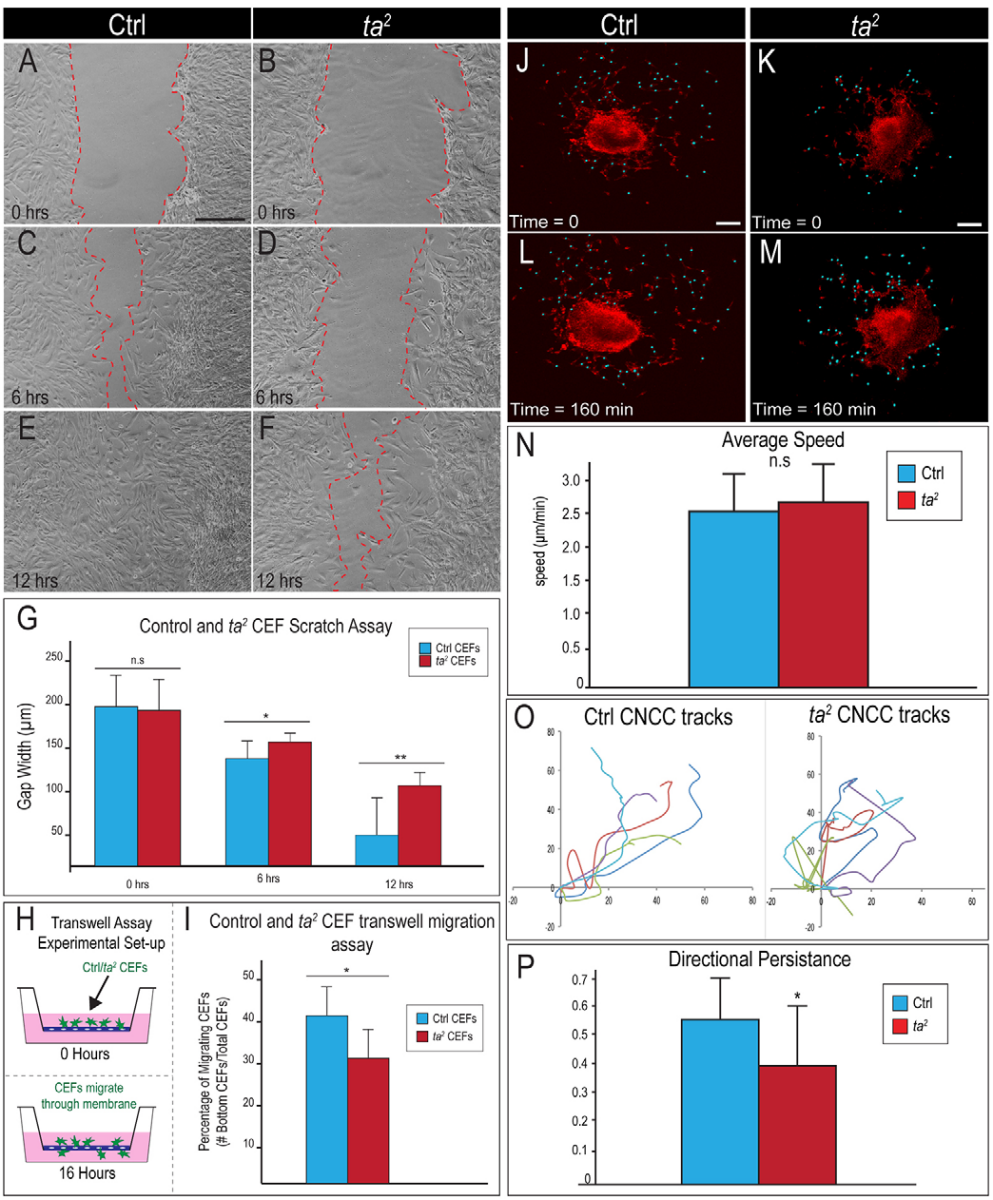
**Loss of C2CD3-dependent ciliogenesis affects cell migration by reducing directional persistence.** (A-F) Scratch assay with control (Ctrl) and *ta^2^* CEFs at the indicated times. (G) Quantification of scratch assays; 0 h, *P*=0.8; 6 h, *P<0.05; 12 h, **P<0.01. (H) Schematic of Transwell-insert assay. (I) Quantification of results from the Transwell-insert assay, *P<0.05. (J-M) Time-lapse analysis of CNCC migration from dorsal neural tube explants. Still images from time-lapse analysis of (J,L) control and (K,M) *ta^2^* explants labeled with di-8-ANEPPS (red; blue dots mark migrating CNCCs). (N) Quantification of the average speed of migration of control and *ta^2^* CNCCs, *P*=0.43. (O) Individual cell migration tracks (different colors) from control and *ta^2^* CNCCs over a 40-min period. (P) Quantification of directional persistence (cell displacement divided by total distance traveled) for control and *ta^2^* CNCCs, *P<0.05. n.s., not significant. Error bars indicate s.d. Scale bars: 100 μm (A, applies to B-F); 100 μm (J,K, apply to L,M).

To functionally test whether loss of C2CD3-dependent ciliogenesis affects the ability of CNCC-derived mesenchyme to migrate, we performed three separate assays – a scratch assay, a Transwell-insert assay and time-lapse imaging of CNCCs migrating from dorsal neural tube explants. First, we isolated CEFs from the developing face of control and *ta^2^* embryos, plated the cells to confluence and scratched through the cellular monolayer. CEF migration into the scratched area was measured at 0, 6, 12 and 24 h (Fig. 4A-F; data not shown). The ability of *ta^2^* CEFs to fill the scratched area was substantially reduced relative to that of control CEFs after 6 h (Fig. 4C,D,G). At 12 h, half of the control CEF experimental replicates had completely filled the scratched area, whereas none of the *ta^2^* CEFs had (Fig. 4E,F,G; control *n*=6, *ta^2^ n*=6). By 24 h, both control and *ta^2^* CEFs had completely filled the scratched area (data not shown). To further assess the migratory ability of cells lacking C2CD3-dependent ciliogenesis, we performed Transwell-insert assays. Control and *ta^2^* CEFs were placed in the upper compartment of the Transwell chamber and challenged to migrate through the pores of the membrane into the lower compartment (Fig. 4H). Over 40% of control CEFs migrated into the lower compartment of the Transwell chamber, whereas only 30% of *ta^2^* CEFs migrated into the lower compartment (Fig. 4I; control *n*=18,320 cells, *ta^2^ n*=18,494 cells). Lastly, to confirm our migratory data from CEFs in CNCCs, we performed time-lapse imaging of dorsal neural tube explants (Fig. 4J-M). Imaris software was used to track both the speed and path of CNCCs migrating away from the explant (Fig. 4N,O). Examination of individual 40-min cell tracks (control *n*=14, *ta^2^ n*=14) revealed that, despite traveling at the same speed (Fig. 4N), control CNCCs moved in a more consistent direction (Fig. 4O). Further analysis determined that control CNCCs maintained a significantly higher level of directional persistence relative to *ta^2^* CNCCs (Fig. 4P). Thus, taken together, our *in vivo, in vitro* and *ex vivo* studies suggest that loss of C2CD3-dependent ciliogenesis negatively impacts the directional migration and persistence of CNCCs.

### C2CD3-dependent ciliogenesis affects CNCC migration in a cell-autonomous manner

**Fig. 5.**
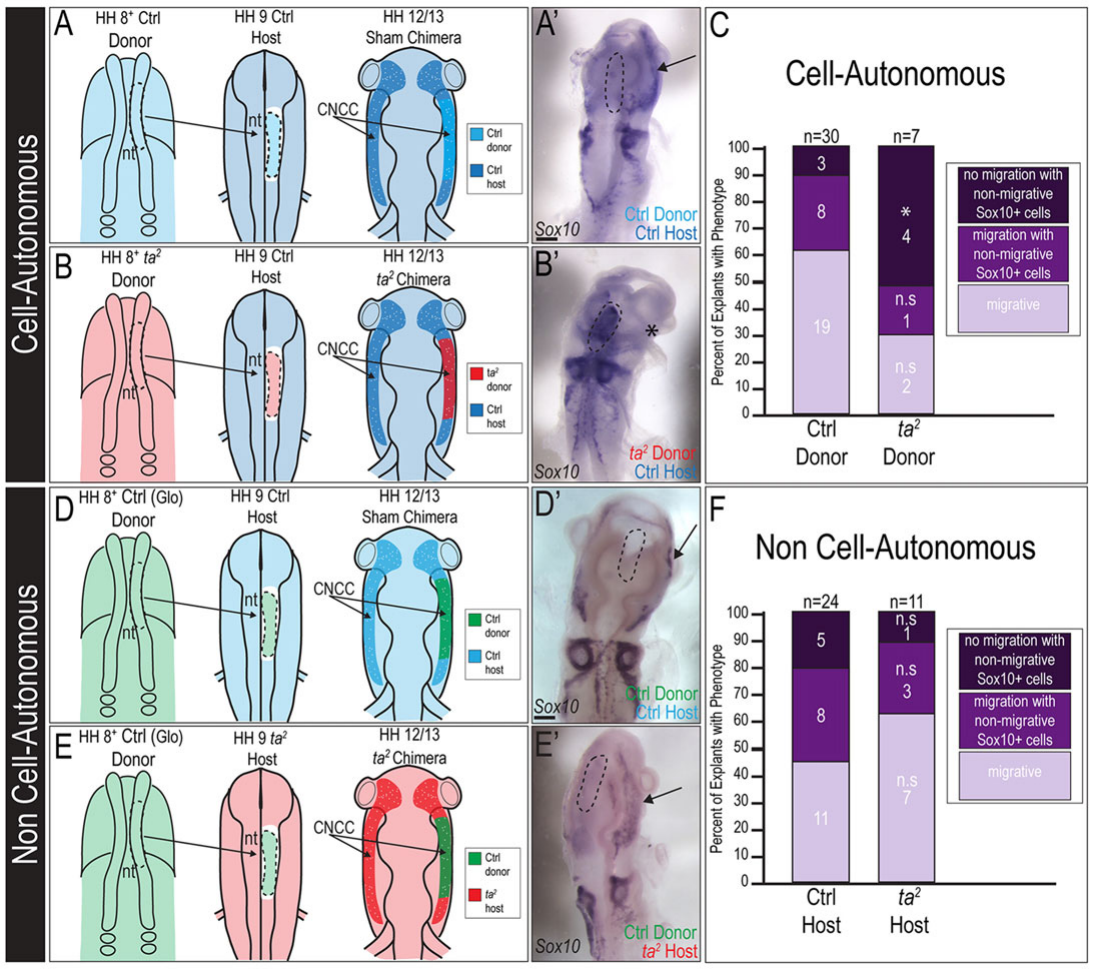
**C2CD3-dependent ciliogenesis affects CNCC migration in a cell-autonomous manner.** (A) Schematic illustration of sham cell-autonomous dorsal neural tube transplant. (A′) *In situ* hybridization for *Sox10* on HH12 sham chimera. The final location of *Sox10-*positive transplanted cells is indicated by a black arrow. (B) Schematic illustration of *ta^2^* cell-autonomous dorsal neural tube transplant. (B′) *In situ* hybridization for *Sox10* on HH12 *ta^2^* chimera [*ta^2^* donor in a control (Ctrl) host]. *Sox10-*positive transplanted cells remain at the original transplant site, rather than migrating laterally (black asterisk). (C) Quantification of cell-autonomous transplant results. Transplants were categorized into three classes – no migration with non-migrating *Sox10*-positive cells (*P<0.01); migration with non-migrating *Sox10*-positive cells (*P*=0.51); migrating (*P*=0.10). (D) Schematic illustration of sham non-cell-autonomous dorsal neural tube transplant. (D′) *In situ* hybridization for *Sox10* on HH12 sham chimera. The final location of *Sox10-*positive transplanted cells is indicated by a black arrow. (E) Schematic illustration of *ta^2^* non-cell-autonomous dorsal neural tube transplant. (E′) *In situ* hybridization for *Sox10* on HH12 *ta^2^* chimera (control donor in a *ta^2^* host). The final location of *Sox10-*positive transplanted cells is indicated by a black arrow. (F) Quantification of non-cell-autonomous transplant results. Transplants were categorized into three classes – no migration with non-migrating *Sox10*-positive cells (*P*=0.41); migration with non-migrating *Sox10*-positive cells (*P*=0.73); migrating (*P*=0.34). (A′,B′,D′,E′) The original transplant site is outlined (dotted black line). Glo, Glo-chick (eGFP-positive tissue); n.s., not significant; nt, neural tube. Scale bars: 150 μm (A′,B′,D′,E′).

CNCC migration requires both intrinsic signaling between CNCCs, and signals from adjacent tissues (e.g. surface ectoderm and mesoderm). Because all tissues in the *ta^2^* mutant exhibited a loss of C2CD3-dependent ciliogenesis, we sought to determine whether aberrant CNCC migration was cell-autonomous or non-cell-autonomous. We performed dorsal neural tube transplants with control and *ta^2^* embryos (Fig. 5A,B,D,E). We first validated our experimental technique of transplantation using a control green fluorescent protein (GFP)-labeled explant (supplementary material Fig. S5). To test whether there were any cell-autonomous mechanisms influencing aberrant CNCC migration, we generated sham and *ta^2^* chimeras, and quantified the percentage of chimeras in which donor CNCCs migrated away from the neural tube (Fig. 5A,A′). In sham chimeras, donor CNCCs migrated away from the dorsal midline completely in 63% of cases (Fig. 5A′,C; *n*=19 out of 30). By contrast, only 28.5% (*n*=2 out of 7) of *ta^2^* chimeras had CNCCs that migrated away from the dorsal midline completely, with the majority of cases (57%, *n*=4 out of 7) having CNCCs that remained at the dorsal midline (Fig. 5B′,C). To test whether there were any non-cell-autonomous mechanisms influencing aberrant CNCC migration in *ta^2^* embryos, we performed reciprocal transplant experiments (Fig. 5D-F). Control CNCCs migrated to equivalent extents in both control and *ta^2^* hosts (Fig. 5F). Taken together, these data suggest that loss of C2CD3-dependent ciliogenesis negatively affects CNCC migration in a cell-autonomous manner.

### C2CD3-dependent ciliogenesis does not affect CNCC proliferation

**Fig. 6.**
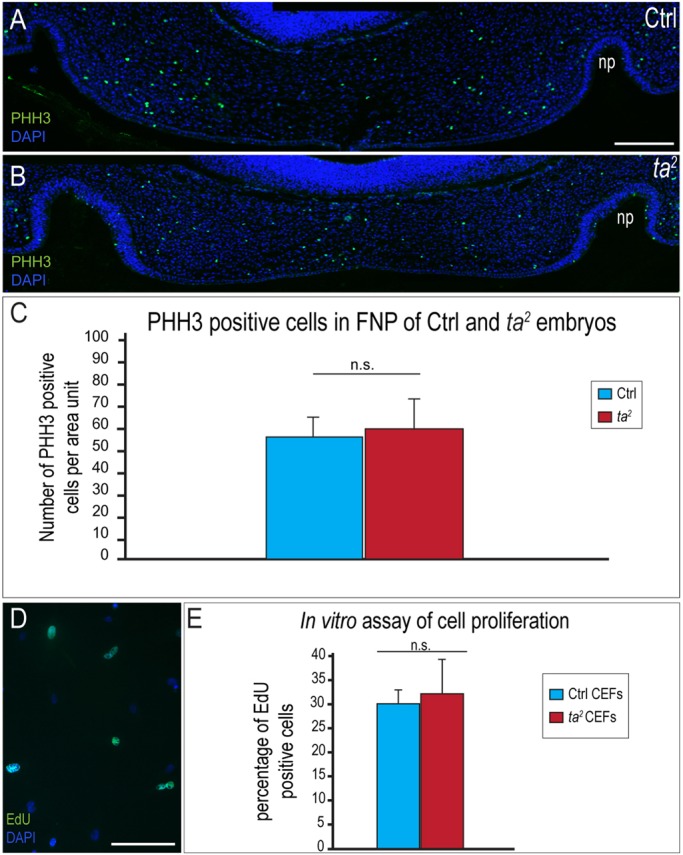
**C2CD3-dependent ciliogenesis does not affect CNCC proliferation.** (A,B) PHH3 immunostaining on frontonasal prominence (FNP) sections of HH22 control (Ctrl) and *ta^2^* embryos. (C) Quantification of PHH3-positive cells normalized over the sample area, *P*=0.48. (D) EdU staining of control CEFs. (E) Quantification of EdU incorporation in control and *ta^2^* CEFs. There is no significant difference (n.s.) in EdU incorporation in *ta^2^* CEFs, *P*=0.14. np, nasal pit. Error bars indicate s.d. Scale bars: 200 μm (A, applies to B); 100 μm (D).

Proliferation is a key event in CNCC development (Brugmann et al., 2007) as appropriate amounts of proliferation determine the shape and mass of the developing prominences of the oral-facial complex. To test whether proliferation is altered when C2CD3-dependent ciliogenesis is lost, we examined control and *ta^2^* embryos and CEFs. Sections from the frontonasal prominence (FNP) of HH22 control and *ta^2^* embryos were immunostained for PHH3, and the number of positive cells was quantified. No significant change in the number of PHH3-positive cells was detected within developing FNPs (Fig. 6A-C; control *n*=17, *ta^2^ n*=14). To confirm this finding, we analyzed control and *ta^2^* CEFs that had been harvested from the facial mesenchyme. CEFs were cultured, pulsed with 5-ethynyl-2′-deoxyuridine (EdU) for 1 h and immunostained (Fig. 6D). There was no significant change in EdU incorporation between control and *ta^2^* CEFs (Fig. 6E; control *n*=11,548 cells, *ta^2^ n*=9792 cells). Taken together, these analyses suggest that loss of C2CD3-dependent ciliogenesis does not cause oral-facial defects by disrupting the rate of CNCC proliferation.

### Defects in C2CD3-dependent ciliogenesis affect cartilage differentiation

**Fig. 7.**
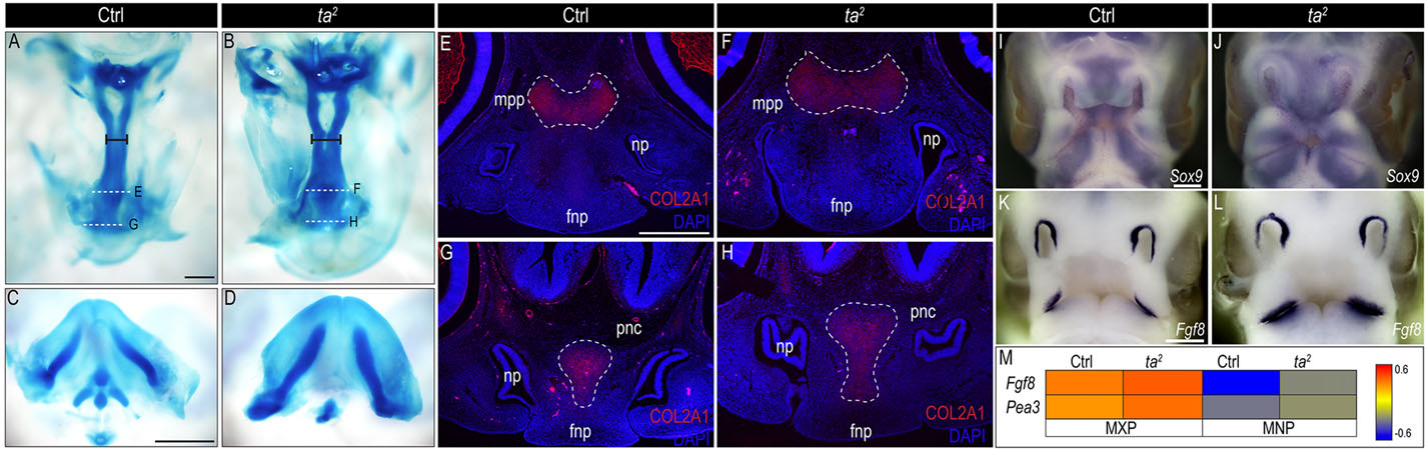
**C2CD3-dependent ciliogenesis affects development of CNCC-derived cartilage.** (A-D) Whole-mount Alcian Blue staining of (A,C) control (Ctrl) and (B,D) *ta^2^* HH28 embryos. (A,B) Ventral view of control and *ta^2^* maxillopalatine processes, black bars represent location at which width measurements were taken. (C,D) Dorsal view of control and *ta^2^* mandibular prominences. (E-H) Immunostaining of COL2A1 (red) in (E,G) control and (F,H) *ta^2^* embryos (plane of section indicated by dotted white lines in A,B). (I-L) Whole-mount *in situ* hybridization for (I,J) *Sox9* and (K,L) *Fgf8* on HH25 control and *ta^2^* embryos. (M) Heatmap of *Fgf8* and *Pea3* expression from RNA-seq analysis of HH25 control and *ta^2^* facial prominences. Fnp, frontonasal prominence; mxp, maxillary prominence; mnp, mandibular prominence; mpp, maxillopalatine process; pnc, prenasal cartilage. Scale bars: 500 μm (A, applies to B; E, applies to F-H); 1000 μm (C, applies to D); 250 μm (I, applies to J); 200 μm (K, applies to L).

OFD-affected individuals and *ta^2^* mutants both have substantial skeletal defects (Abbott et al., 1959, 1960; Gorlin et al., 1990). CNCCs give rise to a significant portion of the skeletal structures in the skull (Noden and Trainor, 2005). We examined the formation of CNCC-derived cartilage in the developing faces of control and *ta^2^* embryos to determine whether C2CD3-dependent ciliogenesis is important for skeletal development. We first examined cranial cartilage through whole-mount Alcian Blue staining at HH28. We observed a statistically significant increase in the width of the maxillopalatine process in the *ta^2^* mutant (Fig. 7A,B; compare length of black bars; control *n*=5, *ta^2^ n*=5; supplementary material Fig. S6A). Additionally, Meckel's cartilage was thickened in *ta^2^* mandibles (Fig. 7C,D). We confirmed the expansion of various facial cartilages in *ta^2^* embryos by examining COL2A1 expression in control and *ta^2^* cranial sections. In addition to observing an expansion of the maxillopalatine process (Fig. 7E,F), we also observed an increase in the size of the prenasal cartilage (Fig. 7G,H). Furthermore, we examined the expression of *Sox9*, a master transcriptional regulator of chondrogenesis (Bi et al., 1999), and found an expanded area of expression throughout *ta^2^* facial prominences (Fig. 7I,J). Taken together, these results suggest that aberrant C2CD3-dependent ciliogenesis results in dysmorphic oral-facial cartilages.

Dysmorphic oral-facial cartilages have been reported in both murine and teleost models of OFD (Ferrante et al., 2009, 2006). The murine model of OFD, as well as another ciliopathic mutant with dysmorphic facial cartilage (Fuz^−/−^), show increased levels of *Ffg8* expression within the developing facial prominences (Tabler et al., 2013). Based on these data, we sought to determine whether *Ffg8* expression was altered in *ta^2^* embryos. By using both *in situ* hybridization and RNA-seq analyses, we detected increased levels of *Fgf8* expression within the facial prominences (Fig. 7K-M). Furthermore, expression of *Pea3*, a key transcriptional target of FGF signaling was also increased in facial prominences (Fig. 7M). These data support a mechanism by which increased levels of *Fgf8* expression could account for increased chondrogenesis in the developing facial prominences of *ta^2^* mutants.

## DISCUSSION

OFD is a ciliopathy that affects development of the face, oral cavity, brain, kidneys and limbs (Annerhn et al., 1984; Baker and Beales, 2009; Zaghloul and Brugmann, 2011). Herein, we propose the avian *ta^2^* mutant as an animal model for OFD based on genetic (Chang et al., 2014), biochemical and phenotypic evidence (Fig. 1). We examined the development of CNCCs in these mutants in order to understand the basis for oral-facial anomalies in cases of human OFD. Our findings suggest that disruption of C2CD3-dependent ciliogenesis does not affect the specification or proliferation of CNCCs, but does cause aberrant CNCC migration and differentiation. Taken together, these data support the use of the *ta^2^* mutant as an animal model that can be utilized to uncover the cellular and molecular etiology of OFD, as well as a system in which to test possible therapeutic treatments.

There are currently 13 different subtypes of OFD, classified by subtleties of phenotypic presentation. The phenotypic features of the various subtypes overlap substantially, and some subtypes are not well defined. Although there is variability in the phenotypic presentation of OFD, most forms of this disorder involve anomalies of the oral cavity, facial features, digits and developing brain. C2CD3-dependent OFD represents the 14th OFD subtype (OMIM 615948) (Thauvin-Robinet et al., 2014), strongly suggesting that the *ta^2^* mutant, previously shown to be caused by a mutation in C2CD3, could be classified as a novel generalized model for OFD, which is particularly relevant to OFD14.

### The biochemical mechanism of C2CD3-dependent OFD is conserved between species

Until recently, only one gene had been exclusively linked to OFD. The *OFD1* gene encodes a ciliary protein that localizes to distal centrioles (Ferrante et al., 2006; Romio et al., 2004; Thauvin-Robinet et al., 2006). Most *OFD1* mutations that have been identified in individuals result in a truncated, non-functional protein (Thauvin-Robinet et al., 2006). A recent analysis of OFD-affected individuals has found that a subset of cases are linked to mutations in C2CD3, a distal centriolar protein that promotes centriole elongation and the recruitment of other distal centriolar proteins, including OFD1 (Thauvin-Robinet et al., 2014; Ye et al., 2014). Data from human and mouse studies show that C2CD3 and OFD1 physically interact to mediate centriolar lengthening and ciliogenesis (Thauvin-Robinet et al., 2014). We found that this interaction was conserved in chicken (Fig. 1). Co-immunoprecipitation experiments showed that avian C2CD3 and OFD1 physically interacted, and that this interaction was negatively affected in *ta^2^* mutants, which produce a truncated form of the C2CD3 protein. Thus, it stands to reason that mutations in C2CD3 and OFD1 generate very similar phenotypes and result in OFD because they comprise a core protein complex at the distal centriole in avian, murine and human cells.

### The role of primary cilia in cell migration, a C2CD3-dependent mechanism

Several ciliary mutants exhibit aberrant cell migration (Osborn et al., 2014; Tabler et al., 2013; Tobin et al., 2008). Fibroblasts from ciliopathic individuals, and fibroblasts and NCCs from ciliopathic animal models fail to migrate normally, exhibiting disruptions in actin cytoskeletal architecture, decreased velocity and decreased directional persistence (Hernandez-Hernandez et al., 2013; Madhivanan et al., 2012; Tobin et al., 2008). The mechanism by which primary cilia contribute to cell migration, however, remains unclear. One potential mechanism is that C2CD3-dependent cell migration involves the localization and/or activation of the GTPases, RhoA and Rac1. RhoA controls many aspects of adhesion and cytoskeletal organization, promoting protrusion collapse. Rac1 is important for lamellipodia stabilization and cell repolarization (Ridley, 2011). RhoA and Rac1 have a mutually antagonistic relationship where activated Rac1 is localized to the migration front, promoting actin polymerization and driving protrusions, and activated RhoA is localized to the trailing edge, driving contraction. Various studies have shown that ciliopathies, such as Lowe, Joubert, and Bardet-Beidl syndromes, have defects in RhoA or Rac1 localization or activity (Hernandez-Hernandez et al., 2013; Madhivanan et al., 2012; Valente et al., 2010). We analyzed Rac1 activation in control and *ta^2^* CEFS and did not detect any substantial difference in the levels of total or activated Rac1 between control and *ta^2^* cells (supplementary material Fig. S7; data not shown). We have yet to analyze RhoA activity in *ta^2^* cells. In light of the fact that RhoA can localize to the centriole and basal body (Valente et al., 2010), it remains possible that RhoA localization and/or activity is perturbed in *ta^2^* cells owing to disruptions in the formation of the protein complex at the distal centriole. Further investigation of RhoA localization and activity in *ta^2^* cells is a focus of our future work.

A second mechanism for aberrant cell migration is based on the cilium functioning as an antenna to detect a chemoattractant gradient. This hypothesis has been supported by the fact that (1) transduction of chemoattractant signals occurs through the primary cilium, (2) primary cilia are required for directed migration of fibroblasts towards a chemoattractant source and (3) receptors for chemoattractants, such as PDGFRα, localize to the axoneme (Schneider et al., 2010, 2005). Specifically, data regarding PDGFRα provide strong support for the existence of this mechanism, as activation of PDGFRα plays dual roles in neural crest cell (NCC) migration by stimulating chemotaxis and regulating cell motility (He and Soriano, 2013; Vasudevan and Soriano, 2014). Loss of PDGFRα in NCCs produces a strikingly similar NCC migration phenotype to that of *ta^2^* mutants*.* NCCs that are unable to respond to PDGFA have migratory defects, a reduced ability to recover from scratch assays and lack directional persistence (He and Soriano, 2013; Vasudevan and Soriano, 2014). Testing the ability of *ta^2^* NCCs to respond to chemotactic gradients is also a focus of our future work.

### The role of C2CD3-dependent ciliogenesis in CNCC differentiation

CNCCs give rise to a wide variety of cell types; however, within the developing face, their major contribution is to the facial skeleton. In *ta^2^* mutants, cartilaginous elements within the oral-facial complex are enlarged and dysmorphic (Fig. 7). Both individuals with ciliopathies and numerous ciliopathic animal models have dysmorphic and/or ectopic facial skeletal elements (Brugmann et al., 2010; Ferrante et al., 2009, 2006; Kjaer et al., 1999; Lunt et al., 2009; Tobin et al., 2008; Weatherbee et al., 2009; Zhang et al., 2011). These data strongly indicate that primary cilia play a role in CNCC differentiation. The mechanism of how primary cilia contribute to this process, however, remains unclear.

Mouse models for OFD, as well as mutants that have similar phenotypes to OFD (Fuz^−/−^), have expanded areas of facial cartilage (as determined by increased expression of *Sox9* and *Col2a1*, and Alcian Blue staining), concordant with increased levels of *Ffg8* expression (Tabler et al., 2013; Zhang et al., 2011). The FGF pathway has previously been implicated in inducing chondrogenesis in the developing facial prominences (Abzhanov and Tabin, 2004). Thus, a possible mechanism explaining enlarged facial cartilage in *ta^2^* mutants (and possibly OFD-affected individuals) is a gain of FGF8 activity. We favor this hypothesis owing to the previously established relationship between *Ffg8* and *Shh*. The growth and development of different craniofacial cartilages in avian embryos has been shown to be positively regulated through the synergistic actions of *Fgf8* and *Shh* (Abzhanov and Tabin, 2004). We have previously reported that Shh activity is increased in *ta^2^* embryos through increased production of GLI3 activator (GLI3A) and reduced production of GLI3 repressor (GLI3R) (Chang et al., 2014). In non-pathological conditions, GLI3R suppresses *Fgf8* expression in the face (Aoto et al., 2002). Thus, in ciliary mutants (including Ofd-1, Fuz^−/−^ and *ta^2^*), increased GLI3A or attenuated GLI3R production could alleviate transcriptional inhibition of *Fgf8* and promote chondrogenesis.

Another hypothesis to explain the production of enlarged areas of cartilage focuses on the role of primary cilia in responding to the range of stimuli that is used to coordinate and regulate cell fate decisions (Irigoin and Badano, 2011). Primary cilia extend from human embryonic stem cells (Kiprilov et al., 2008), and there is precedence for the loss of cilia inherently changing the potentiality of stem cells and other multipotent cell types (Huang et al., 2014; Hunkapiller et al., 2011). Lineage experiments on cephalic neural crest stem cells support the notion of a hierarchical model in which lineage decisions are generated through progressive restrictions in the potentialities of a highly multipotent progenitor CNCC that can give rise to specific percentages of neural and mesenchymal (i.e. cartilage) derivatives (Le Douarin et al., 2008). In the absence of Shh, the majority of derivatives from clonal culture were ‘neural only’, with a smaller population giving rise to ‘mesenchymal or neural’ derivatives, and an even smaller population giving rise to ‘mesenchymal only’ derivatives. Treatment with Shh increases the percentage of clones exhibiting ‘mesenchymal or neural’ and ‘mesenchymal only’ potentialities, as well as decreasing the frequency of the ‘neural only’ colonies (Le Douarin et al., 2012). Thus, a second possible mechanism to explain enlarged areas of cartilage in *ta^2^* mutants, and possibly OFD-affected individuals, is that the C2CD3-dependent loss of cilia leads to altered CNCC progenitor potential through increased responsiveness to Shh. Understanding how loss of cilia affects the potentialities of CNCCs is the focus of our ongoing and future work.

In summary, genetic findings from our previous work (Chang et al., 2014) coupled with phenotypic and biochemical analyses performed herein, provide the first direct evidence that the *ta^2^* mutant can serve as a bona fide model of human OFD. In light of these findings, we have used the *ta^2^* model to examine the possible cellular etiology of the oral-facial phenotypes of human OFD. We focused specifically on CNCC development in this disease process because these cells make essential contributions to the majority of the oral-facial domains that are affected in OFD. We confirmed that CNCCs extend primary cilia during all ontogenic phases, and that loss of C2CD3-dependent ciliogenesis affects CNCC migration and differentiation, but not specification or proliferation. Based on these observations, we speculate that disrupted migration, aberrant molecular signaling and/or altered CNCC potentialities could contribute to the oral-facial phenotypes in human cases of OFD. With the knowledge of specific C2CD3 mutations in humans (Thauvin-Robinet et al., 2014) and the understanding of the role of C2CD3 in the primary cilia (Ye et al., 2014), our future work will focus on understanding the molecular mechanisms of C2CD3-mediated CNCC migration and differentiation, in hopes of uncovering avenues for therapeutic intervention.

## MATERIALS AND METHODS

### Avian embryo preparation

Control and *ta^2^* eggs were incubated at 39°C until they reached the desired Hamburger and Hamilton stage (Hamburger and Hamilton, 1951); they were then harvested for analysis.

### Embryo genotyping

Embryos younger than HH25 were genotyped as previously described (Chang et al., 2014).

### Hematoxylin and eosin staining

Sections were deparaffinized and rehydrated, and nuclei were stained with hematoxylin (Polysciences, Warrington, PA, USA). Sections were rinsed in water and then placed briefly in eosin Y (Sigma-Aldrich, St Louis, MO, USA). Sections were dehydrated and mounted using Permount (Fisher Scientific, Waltham, MA, USA).

### Micro-computed tomography

Embryos at day 13 were harvested and fixed overnight in 4% PFA, washed in PBS and placed in 50% Lugol solution (L6146-1L, Sigma-Aldrich, St Louis, MO, USA) for 2 weeks, with frequent refreshing. Heads were scanned using MicroCAT II v. 1.9d (Imtek) with COBRA v.7.4 (Exxim Computing Corporation) software used for image reconstruction. OsiriX was used for image display and analysis.

### Isolation of CEFs

Facial prominences and limb buds were collected from HH25 control or *ta^2^* embryos and digested in PBS (pH 7.4) with 1 mg/ml collagenase (Roche, Indianapolis, IN, USA) at 37°C for 30 min. Cells were dissociated by gentle pipetting and collected using centrifugation. The medium contained Dulbecco's modified Eagle's medium (DMEM; Life Technologies, Grand Island, NY, USA), 10% fetal bovine serum (Fisher, Waltham, MA, USA) and 50 U/ml penicillin-streptomycin (Life Technologies, Grand Island, NY, USA). CEFs from passage 5-15 were used for experiments.

### CEF transfection

CEFs at 90% confluence were transfected with p3×FLAG-*C2cd3^Ctrl^*, p3×FLAG-*C2cd3^ta2^* and pcDNA3.1-*Ofd1*-Myc (2.5 µg per 60 mm dish) and harvested after 24 h for co-immunoprecipitation experiments using a transfection reagent (X-tremeGENE, Roche, Indianapolis, IN, USA).

### Co-immunoprecipitation and western blot analyses

Lysates were prepared from transfected CEFs and incubated with a monoclonal antibody against FLAG (1:1000; M2, F1804, Sigma-Aldrich, St Louis, MO, USA). Dynabeads protein G was added to pull down 3×FLAG-C2cd3 protein. Beads were washed and boiled in 1× Laemmli sample buffer for 3 min. Proteins were separated on 8% SDS-PAGE (for OFD1-Myc) or 6% SDS-PAGE (for 3×FLAG-*C2cd3^Ctrl^* or 3×FLAG-*C2cd3^ta2^*) and transferred to PVDF membrane. The membranes were blocked in 6% non-fat milk in 1× TBST for 20 min at 4°C. Western blotting was used to analyze the immunoprecipitated proteins by using primary monoclonal antibodies against FLAG or Myc (9B11, Cell Signaling, Beverly, MA, USA). Horseradish peroxidase (HRP)-conjugated (goat) anti-mouse (Santa Cruz Biotechnology, Santa Cruz, CA, USA) was used as the secondary antibody. An electrochemiluminescence (ECL) assay (ECL prime, Amersham, Pittsburg, PA, USA) was performed to develop the chemiluminescence signals. ImageJ software was used to quantify the signals on the radiographic films (CL-XPosure film, Thermo Scientific, Waltham, MA, USA) as follows – the peak percentage of OFD1 proteins (co-immunoprecipitation) was normalized to that of C2CD3 proteins (immunoprecipitation), and then the relative OFD1 precipitation was calculated by the ratio of OFD1/C2CD3*^ta2^* to OFD1/C2CD3^Ctrl^.

### Immunohistochemistry

Immunostaining was performed according to standard protocols. Briefly, embryos were fixed in DENT solution or 4% PFA. Sections were incubated in primary antibody for 1 h at room temperature or overnight at 4°C. Secondary antibodies with fluorescent tags were then applied at a dilution of 1:1000 and incubated at room temperature for 1 h. Slides were stained with 4′,6-diamino-2-phenylindone (DAPI; 1:10,000; Life Technologies, Grand Island, NY, USA) and mounted with mounting medium (ProLong Gold, Life Technologies, Grand Island, NY, USA). For whole-mount immunostaining, embryos were blocked overnight at 4°C and then incubated in primary antibody overnight. Embryos were incubated in secondary antibodies (1:500) overnight at 4°C and DAPI (1:10,000). Embryos were cleared with 50% glycerol and then mounted in 70% glycerol. Antibodies used in this study were against glutamylated-tubulin (rabbit; 1:500; AB3201, Millipore, Billerica, MA, USA), PAX7 (rabbit; 1:20; Developmental Studies Hybridoma Bank, Iowa City, IA, USA), HNK1 (hybridoma; 1:20; Developmental Studies Hybridoma Bank, Iowa City, IA, USA), PHH3 (mouse; 1:1000; 05-1336, Millipore, Billerica, MA, USA), PCNA (mouse; 1:1000; 2586, Cell Signaling Technology, Danvers, MA, USA) and COL2A1 (mouse; 1:500; MAB8887, Millipore, Billerica, MA, USA).

### Delaunay triangulation

HNK1-stained sections were imaged using a Leica DM5000 B microscope, and files were analyzed in Photoshop. Nuclei of HNK1-positive cells (migrating NCCs) were marked, and spatial distribution was analyzed bilaterally using the Delaunay–Voronoi algorithm (ImageJ). The coordinates of all line segments were exported for further analysis. Using MATLAB, vertices of triangles created by the Delaunay–Voronoi algorithm were identified. Coordinates were used to determine the area of each triangle.

### qRT-PCR

HH10 embryos were harvested and the anterior portion of the embryos was retained for qRT-PCR analyses of *Sox10*. RNA was extracted using TRIzol reagent (Life Technologies, Grand Island, NY, USA), and cDNA was generated using SuperScript III (Life Technologies, Grand Island, NY, USA). SsoAdvanced SYBR Green Supermix (Bio-Rad, Hercules, CA, USA) and a CFX96 Touch real-time PCR detection system (Bio-Rad, Hercules, CA, USA) were used to perform qRT-PCR. *Sox10* expression was normalized to *Gapdh* expression. Student's *t*-test was used for statistical analysis. Primers – *Gapdh*-F, 5ʹ-CAACATCAAATGGGCAGATG-3ʹ; *Gapdh*-R, 5ʹ-AGCTGAGGGAGCTGAGATGA-3ʹ; *Sox10*-F, 5ʹ-AACGCCTTCATGGTCTGG-3ʹ; *Sox10*-R, 5ʹ-GGGACGCTTATCACTTTCATTC-3ʹ.

### TUNEL staining

The TUNEL assay was performed using the *In Situ* Cell Death Detection Kit, Fluorescein according to the manufacturer's protocol (Ref, 11 684 795 910, Roche, Indianapolis, IN, USA). For whole-mount TUNEL staining, an anti-fluorescein antibody, Fab fragment from sheep, conjugated with alkaline phosphatase (AP) (Ref, 11 772 457 001, Roche, Indianapolis, IN, USA) was applied, and the staining of embryos was developed in NBT/BCIP substrate solution (Amresco, Solon, OH, USA).

### Scratch assay

Control and *ta^2^* CEFs were seeded in 12-well plates and allowed to recover overnight in DMEM GlutaMAX medium (Life Technologies, Grand Island, NY, USA) with 10% sheep serum (Fisher, Waltham, MA, USA). Once confluent, a pipette tip was used to scratch through the cellular monolayer. Wells were scratched twice so that a cross was formed in order to create a point of reference for imaging. Cells were briefly washed to remove debris and then immediately imaged. Plates were returned to the incubator and imaged at 6, 12 and 24 h following injury.

### Transwell assay

Control or *ta^2^* CEFs (2×10^4^) were seeded onto FluoroBlok cell culture inserts with 8-μm pores (351157, Corning, Tewksbury, MA, USA) and left to migrate overnight (16 h). Inserts were fixed and stained with Hoechst 33342. Six representative fields from the top and bottom of the membrane were imaged. Nuclei were counted, and statistical analysis was performed.

### Time-lapse imaging

Dorsal neural tubes were harvested from HH8^+^- to HH9-control and -*ta^2^* embryos, and cultured in neural crest cell medium (Bajpai et al., 2010) in an 8-well chamber slide (μ–slide 8 well, 80821, Ibidi, Verona, WI, USA) that had been coated with fibronectin. The remaining portion of the embryo was used for genotyping. Explants were incubated at 37°C for 5-6 h. Approximately 30 min before imaging, neural crest cell medium was replaced with 3 μM Di-8-Anneps (a gift from Brian Sirosky, CCHMC, Cincinnati, OH, USA) in order to fluorescently label cell membranes. Explants were imaged for 12 h at 2-min intervals using a Nikon A1Rsi inverted confocal microscope. Videos were analyzed using Imaris software.

### Transplant assay

Control and *ta^2^* eggs were windowed at HH8 to HH9. The vitelline membrane was removed near the anterior portion of the host embryo using tungsten needles. Tyrode's solution (Ca^2+^ and Mg^2+^ free) was added to the egg, and the right side of the dorsal neural tube from the mesencephalon to the second rhombomere was removed. The host egg was briefly set aside as the donor was prepared. HH8^+^ control or *ta^2^* embryos were harvested and placed in Tyrode's solution. An equivalent portion of the right dorsal neural tube was removed from the donor and transferred to the host egg. The donor explant was placed into the ablation site. Transplanted embryos recovered at room temperature for 10 min, were sealed with tape and returned to the incubator. The remaining portion of the donor embryos was used for genotyping. Following overnight incubation, the transplanted embryos were examined for a heartbeat, and embryos that survived were harvested and fixed in 4% PFA for analysis.

### *In situ* hybridization

Patterns of gene expression in control and *ta^2^* embryos were analyzed by using whole-mount *in situ* hybridization with digoxigenin-labeled riboprobes, as described on the gallus expression *in situ* hybridization analysis site (GEISHA) (Bell et al., 2004; Darnell et al., 2007). Probes for *Pax7*, *Sox10*, *Snai2*, *Sox9* and *Fgf8* were designed according to sequences listed on GEISHA (Darnell et al., 2007).

### EdU staining

Control and *ta^2^* CEFs (1×10^4^) were plated on coverslips that had been coated with fibronectin (BD Biosciences, San Jose, CA, USA); they were then left to recover overnight. CEFs were pulsed with 10 μM EdU for 1 h and then fixed for 15 min with 3.7% formaldehyde. EdU staining was performed according to the manufacturer's protocol (Click-IT EdU Alexa Flour 488 Imaging Kit; Life Technologies, Eugene, OR, USA).

### Alcian Blue

Embryos were fixed in Bouin's solution overnight and then washed with 70% EtOH and 0.1% NH_4_OH solution, equilibrated in 5% acetic acid and stained with 0.05% Alcian Blue 8GX (Fisher Scientific, Waltham, MA, USA) in 5% acetic acid for 4 h. Embryos were washed with 5% acetic acid followed by 100% MeOH. Benzyl alcohol (benzylbenzoate) (1:2) was used to clear the embryos.

### RNA-seq

RNA-seq and data analysis was performed as described previously (Chang et al., 2014).

### Rac1 activity

Rac1 levels and activity were determined using the Active Rac1 Detection Kit (8851, Cell Signaling, Beverly, MA, USA), as per the manufacturer's protocol.

## Supplementary Material

Supplementary Material

## References

[DMM020222C1] Abbott U. K., Taylor L. W. and Abplanalp H. (1959). A second talpid-like mutation in the fowl. *Poul. Sci.* 38, 1185.

[DMM020222C2] Abbott U. K., Taylor L. W. and Abplanalp H. (1960). Studies with *talpid*^2^, an embryonic lethal of the fowl. *J. Hered.* 51, 195-202.

[DMM020222C3] Abzhanov A. and Tabin C. J. (2004). Shh and Fgf8 act synergistically to drive cartilage outgrowth during cranial development. *Dev. Biol.* 273, 134-148. 10.1016/j.ydbio.2004.05.028 15302603

[DMM020222C4] Annerhn G., Arvidson B., Gustavson K.-H., Jorulf H. and Carlsson G. (1984). Oro-facio-digital syndromes I and II: radiological methods for diagnosis and the clinical variations. *Clin. Genet.* 26, 178-186. 10.1111/j.1399-0004.1984.tb04365.x 6478638

[DMM020222C5] Aoto K., Nishimura T., Eto K. and Motoyama J. (2002). Mouse GLI3 regulates Fgf8 expression and apoptosis in the developing neural tube, face, and limb bud. *Dev. Biol.* 251, 320-332. 10.1006/dbio.2002.0811 12435361

[DMM020222C6] Bajpai R., Chen D. A., Rada-Iglesias A., Zhang J., Xiong Y., Helms J., Chang C.-P., Zhao Y., Swigut T. and Wysocka J. (2010). CHD7 cooperates with PBAF to control multipotent neural crest formation. *Nature* 463, 958-962. 10.1038/nature08733 20130577 PMC2890258

[DMM020222C7] Baker K. and Beales P. L. (2009). Making sense of cilia in disease: the human ciliopathies. *Am. J. Med. Genet. C Semin. Med. Genet.* 151C, 281-295. 10.1002/ajmg.c.30231 19876933

[DMM020222C8] Beales P. L., Elcioglu N., Woolf A. S., Parker D. and Flinter F. A. (1999). New criteria for improved diagnosis of Bardet-Biedl syndrome: results of a population survey. *J. Med. Genet.* 36, 437-446. 10874630 PMC1734378

[DMM020222C65] Bell G. W., Yatskievych T. A. and Antin P. B. (2004). GEISHA, a whole-mount in situ hybridization gene expression screen in chicken embryos. *Dev. Dyn.* 229, 677-687. 10.1002/dvdy.10503 14991723

[DMM020222C9] Bi W., Deng J. M., Zhang Z., Behringer R. R. and de Crombrugghe B. (1999). Sox9 is required for cartilage formation. *Nat. Genet.* 22, 85-89. 10.1038/8792 10319868

[DMM020222C10] Brugmann S. A., Goodnough L. H., Gregorieff A., Leucht P., ten Berge D., Fuerer C., Clevers H., Nusse R. and Helms J. A. (2007). Wnt signaling mediates regional specification in the vertebrate face. *Development* 134, 3283-3295. 10.1242/dev.005132 17699607

[DMM020222C11] Brugmann S. A., Allen N. C., James A. W., Mekonnen Z., Madan E. and Helms J. A. (2010). A primary cilia-dependent etiology for midline facial disorders. *Hum. Mol. Genet.* 19, 1577-1592. 10.1093/hmg/ddq030 20106874 PMC2846163

[DMM020222C12] Carmona-Fontaine C., Theveneau E., Tzekou A., Tada M., Woods M., Page K. M., Parsons M., Lambris J. D. and Mayor R. (2011). Complement fragment C3a controls mutual cell attraction during collective cell migration. *Dev. Cell* 21, 1026-1037. 10.1016/j.devcel.2011.10.012 22118769 PMC3272547

[DMM020222C13] Chai Y., Jiang X., Ito Y., Bringas P. Jr, Han J., Rowitch D. H., Soriano P., McMahon A. P. and Sucov H. M. (2000). Fate of the mammalian cranial neural crest during tooth and mandibular morphogenesis. *Development* 127, 1671-1679. 10725243 10.1242/dev.127.8.1671

[DMM020222C14] Chang C.-F., Schock E. N., O'Hare E. A., Dodgson J., Cheng H. H., Muir W. M., Edelmann R. E., Delany M. E. and Brugmann S. A. (2014). The cellular and molecular etiology of the craniofacial defects in the avian ciliopathic mutant talpid2. *Development* 141, 3003-3012. 10.1242/dev.105924 25053433 PMC4197679

[DMM020222C15] Darnell D. K., Kaur S., Stanislaw S., Davey S., Konieczka J. H., Yatskievych T. A. and Antin P. B. (2007). GEISHA: an in situ hybridization gene expression resource for the chicken embryo. *Cytogenet. Genome Res.* 117, 30-35. 10.1159/000103162 17675842

[DMM020222C16] Dixon J., Jones N. C., Sandell L. L., Jayasinghe S. M., Crane J., Rey J.-P., Dixon M. J. and Trainor P. A. (2006). Tcof1/Treacle is required for neural crest cell formation and proliferation deficiencies that cause craniofacial abnormalities. *Proc. Natl. Acad. Sci. USA* 103, 13403-13408. 10.1073/pnas.0603730103 16938878 PMC1557391

[DMM020222C17] Dvorak L. and Fallon J. F. (1991). Talpid2 mutant chick limb has anteroposterior polarity and altered patterns of programmed cell death. *Anat. Rec.* 231, 251-260. 10.1002/ar.1092310213 1746725

[DMM020222C18] Ferrante M. I., Feather S. A., Bulfone A., Wright V., Ghiani M., Selicorni A., Gammaro L., Scolari F., Woolf A. S., Sylvie O. et al. (2001). Identification of the gene for oral-facial-digital type I syndrome. *Am. J. Hum. Genet.* 68, 569-576. 10.1086/318802 11179005 PMC1274470

[DMM020222C19] Ferrante M. I., Barra A., Truong J.-P., Banfi S., Disteche C. M. and Franco B. (2003). Characterization of the OFD1/Ofd1 genes on the human and mouse sex chromosomes and exclusion of Ofd1 for the Xpl mouse mutant. *Genomics* 81, 560-569. 10.1016/S0888-7543(03)00091-0 12782125

[DMM020222C20] Ferrante M. I., Zullo A., Barra A., Bimonte S., Messaddeq N., Studer M., Dollé P. and Franco B. (2006). Oral-facial-digital type I protein is required for primary cilia formation and left-right axis specification. *Nat. Genet.* 38, 112-117. 10.1038/ng1684 16311594

[DMM020222C21] Ferrante M. I., Romio L., Castro S., Collins J. E., Goulding D. A., Stemple D. L., Woolf A. S. and Wilson S. W. (2009). Convergent extension movements and ciliary function are mediated by ofd1, a zebrafish orthologue of the human oral-facial-digital type 1 syndrome gene. *Hum. Mol. Genet.* 18, 289-303. 10.1093/hmg/ddn356 18971206 PMC2638777

[DMM020222C22] Fraser F. C. and Lytwyn A. (1981). Spectrum of anomalies in the Meckel syndrome, or: “maybe there is a malformation syndrome with at least one constant anomaly”. *Am. J. Med. Genet.* 9, 67-73. 10.1002/ajmg.1320090112 7246621

[DMM020222C23] Gorlin R. J., Cohen M. M. and Levin L. S. (1990). *Syndromes of the Head and Neck*. New York: Oxford University Press.

[DMM020222C24] Hamburger V. and Hamilton H. L. (1951). A series of normal stages in the development of the chick embryo. *J. Morphol.* 88, 49-92. 10.1002/jmor.1050880104 24539719

[DMM020222C25] Harris M. P., Hasso S. M., Ferguson M. W. J. and Fallon J. F. (2006). The development of archosaurian first-generation teeth in a chicken mutant. *Curr. Biol.* 16, 371-377. 10.1016/j.cub.2005.12.047 16488870

[DMM020222C26] He F. and Soriano P. (2013). A critical role for PDGFRalpha signaling in medial nasal process development. *PLoS Genet.* 9, e1003851 10.1371/journal.pgen.1003851 24086166 PMC3784569

[DMM020222C27] Hernandez-Hernandez V., Pravincumar P., Diaz-Font A., May-Simera H., Jenkins D., Knight M. and Beales P. L. (2013). Bardet-Biedl syndrome proteins control the cilia length through regulation of actin polymerization. *Hum. Mol. Genet.* 22, 3858-3868. 10.1093/hmg/ddt241 23716571 PMC3766180

[DMM020222C28] Hoover A. N., Wynkoop A., Zeng H., Jia J., Niswander L. A. and Liu A. (2008). C2cd3 is required for cilia formation and Hedgehog signaling in mouse. *Development* 135, 4049-4058. 10.1242/dev.029835 19004860 PMC3120044

[DMM020222C29] Huang J.-G., Shen C.-B., Wu W.-B., Ren J.-W., Xu L., Liu S. and Yang Q. (2014). Primary cilia mediate sonic hedgehog signaling to regulate neuronal-like differentiation of bone mesenchymal stem cells for resveratrol induction in vitro. *J. Neurosci. Res.* 92, 587-596. 10.1002/jnr.23343 24464877

[DMM020222C30] Hunkapiller J., Singla V., Seol A. and Reiter J. F. (2011). The ciliogenic protein Oral-Facial-Digital 1 regulates the neuronal differentiation of embryonic stem cells. *Stem Cells Dev.* 20, 831-841. 10.1089/scd.2010.0362 20873986 PMC3128778

[DMM020222C31] Irigoin F. and Badano J. L. (2011). Keeping the balance between proliferation and differentiation: the primary cilium. *Curr. Genomics* 12, 285-297. 10.2174/138920211795860134 22131874 PMC3131736

[DMM020222C32] Kiprilov E. N., Awan A., Desprat R., Velho M., Clement C. A., Byskov A. G., Andersen C. Y., Satir P., Bouhassira E. E., Christensen S. T. et al. (2008). Human embryonic stem cells in culture possess primary cilia with hedgehog signaling machinery. *J. Cell Biol.* 180, 897-904. 10.1083/jcb.200706028 18332216 PMC2265400

[DMM020222C33] Kjaer K. W., Hansen B. F., Keeling J. W., Nolting D. and Kjaer I. (1999). Malformations of cranial base structures and pituitary gland in prenatal Meckel syndrome. *APMIS* 107, 937-944. 10.1111/j.1699-0463.1999.tb01494.x 10549591

[DMM020222C34] Kuo B. R. and Erickson C. A. (2010). Regional differences in neural crest morphogenesis. *Cell Adh. Migr.* 4, 567-585. 10.4161/cam.4.4.12890 20962585 PMC3011260

[DMM020222C35] Le Douarin N. M. and Dupin E. (1993). Cell lineage analysis in neural crest ontogeny. *J. Neurobiol.* 24, 146-161. 10.1002/neu.480240203 8445384

[DMM020222C36] Le Douarin N. M., Creuzet S., Couly G. and Dupin E. (2004). Neural crest cell plasticity and its limits. *Development* 131, 4637-4650. 10.1242/dev.01350 15358668

[DMM020222C37] Le Douarin N. M., Calloni G. W. and Dupin E. (2008). The stem cells of the neural crest. *Cell Cycle* 7, 1013-1019. 10.4161/cc.7.8.5641 18414040

[DMM020222C38] Le Douarin N. M., Couly G. and Creuzet S. E. (2012). The neural crest is a powerful regulator of pre-otic brain development. *Dev. Biol.* 366, 74-82. 10.1016/j.ydbio.2012.01.007 22269168

[DMM020222C39] Lorda-Sanchez I., Ayuso C., Sanz R. and Ibañez A. (2001). Does Bardet-Biedl syndrome have a characteristic face? *J. Med. Genet.* 38, e14 10.1136/jmg.38.5.e14 11333870 PMC1734874

[DMM020222C40] Lunt S. C., Haynes T. and Perkins B. D. (2009). Zebrafish ift57, ift88, and ift172 intraflagellar transport mutants disrupt cilia but do not affect hedgehog signaling. *Dev. Dyn.* 238, 1744-1759. 10.1002/dvdy.21999 19517571 PMC2771627

[DMM020222C41] Madhivanan K., Mukherjee D. and Aguilar R. C. (2012). Lowe syndrome: between primary cilia assembly and Rac1-mediated membrane remodeling. *Commun. Integr. Biol.* 5, 641-644. 10.4161/cib.21952 23739214 PMC3541337

[DMM020222C42] Maria B. L., Boltshauser E., Palmer S. C. and Tran T. X. (1999). Clinical features and revised diagnostic criteria in Joubert syndrome. *J. Child Neurol.* 14, 583-590; discussion 590-591 10.1177/088307389901400906 10488903

[DMM020222C43] Mayor R. and Carmona-Fontaine C. (2010). Keeping in touch with contact inhibition of locomotion. *Trends Cell Biol.* 20, 319-328. 10.1016/j.tcb.2010.03.005 20399659 PMC2927909

[DMM020222C44] Minoux M. and Rijli F. M. (2010). Molecular mechanisms of cranial neural crest cell migration and patterning in craniofacial development. *Development* 137, 2605-2621. 10.1242/dev.040048 20663816

[DMM020222C45] Noden D. M. and Trainor P. A. (2005). Relations and interactions between cranial mesoderm and neural crest populations. *J. Anat.* 207, 575-601. 10.1111/j.1469-7580.2005.00473.x 16313393 PMC1571569

[DMM020222C46] Osborn D. P. S., Roccasecca R. M., McMurray F., Hernandez-Hernandez V., Mukherjee S., Barroso I., Stemple D., Cox R., Beales P. L. and Christou-Savina S. (2014). Loss of FTO antagonises Wnt signaling and leads to developmental defects associated with ciliopathies. *PLoS ONE* 9, e87662 10.1371/journal.pone.0087662 24503721 PMC3913654

[DMM020222C47] Poretti A., Brehmer U., Scheer I., Bernet V. and Boltshauser E. (2008). Prenatal and neonatal MR imaging findings in oral-facial-digital syndrome type VI. *AJNR Am. J. Neuroradiol.* 29, 1090-1091. 10.3174/ajnr.A1038 18356465 PMC8118817

[DMM020222C48] Ridley A. J. (2011). Life at the leading edge. *Cell* 145, 1012-1022. 10.1016/j.cell.2011.06.010 21703446

[DMM020222C49] Romio L., Fry A. M., Winyard P. J. D., Malcolm S., Woolf A. S. and Feather S. A. (2004). OFD1 is a centrosomal/basal body protein expressed during mesenchymal-epithelial transition in human nephrogenesis. *J. Am. Soc. Nephrol.* 15, 2556-2568. 10.1097/01.ASN.0000140220.46477.5C 15466260

[DMM020222C50] Schneider R. A., Hu D. and Helms J. A. (1999). From head to toe: conservation of molecular signals regulating limb and craniofacial morphogenesis. *Cell Tissue Res.* 296, 103-109. 10.1007/s004410051271 10199970

[DMM020222C51] Schneider L., Clement C. A., Teilmann S. C., Pazour G. J., Hoffmann E. K., Satir P. and Christensen S. T. (2005). PDGFRαα signaling is regulated through the primary cilium in fibroblasts. *Curr. Biol.* 15, 1861-1866. 10.1016/j.cub.2005.09.012 16243034

[DMM020222C52] Schneider L., Cammer M., Lehman J., Nielsen S. K., Guerra C. F., Veland I. R., Stock C., Hoffmann E. K., Yoder B. K., Schwab A. et al. (2010). Directional cell migration and chemotaxis in wound healing response to PDGF-AA are coordinated by the primary cilium in fibroblasts. *Cell Physiol. Biochem.* 25, 279-292. 10.1159/000276562 20110689 PMC2924811

[DMM020222C53] Singla V., Romaguera-Ros M., Garcia-Verdugo J. M. and Reiter J. F. (2010). Ofd1, a human disease gene, regulates the length and distal structure of centrioles. *Dev. Cell* 18, 410-424. 10.1016/j.devcel.2009.12.022 20230748 PMC2841064

[DMM020222C54] Tabler J. M., Barrell W. B., Szabo-Rogers H. L., Healy C., Yeung Y., Perdiguero E. G., Schulz C., Yannakoudakis B. Z., Mesbahi A., Wlodarczyk B. et al. (2013). Fuz mutant mice reveal shared mechanisms between ciliopathies and FGF-related syndromes. *Dev. Cell* 25, 623-635. 10.1016/j.devcel.2013.05.021 23806618 PMC3697100

[DMM020222C55] Thauvin-Robinet C., Cossee M., Cormier-Daire V., Van Maldergem L., Toutain A., Alembik Y., Bieth E., Layet V., Parent P., David A. et al. (2006). Clinical, molecular, and genotype-phenotype correlation studies from 25 cases of oral-facial-digital syndrome type 1: a French and Belgian collaborative study. *J. Med. Genet.* 43, 54-61. 10.1136/jmg.2004.027672 16397067 PMC2564504

[DMM020222C56] Thauvin-Robinet C., Lee J. S., Lopez E., Herranz-Perez V., Shida T., Franco B., Jego L., Ye F., Pasquier L., Loget P. et al. (2014). The oral-facial-digital syndrome gene C2CD3 encodes a positive regulator of centriole elongation. *Nat. Genet.* 46, 905-911. 10.1038/ng.3031 24997988 PMC4120243

[DMM020222C57] Tobin J. L., Di Franco M., Eichers E., May-Simera H., Garcia M., Yan J., Quinlan R., Justice M. J., Hennekam R. C., Briscoe J. et al. (2008). Inhibition of neural crest migration underlies craniofacial dysmorphology and Hirschsprung's disease in Bardet-Biedl syndrome. *Proc. Natl. Acad. Sci. USA* 105, 6714-6719. 10.1073/pnas.0707057105 18443298 PMC2373327

[DMM020222C58] Valente E. M., Logan C. V., Mougou-Zerelli S., Lee J. H., Silhavy J. L., Brancati F., Iannicelli M., Travaglini L., Romani S., Illi B. et al. (2010). Mutations in TMEM216 perturb ciliogenesis and cause Joubert, Meckel and related syndromes. *Nat. Genet.* 42, 619-625. 10.1038/ng.594 20512146 PMC2894012

[DMM020222C59] Vasudevan H. N. and Soriano P. (2014). SRF regulates craniofacial development through selective recruitment of MRTF cofactors by PDGF signaling. *Dev. Cell* 31, 332-344. 10.1016/j.devcel.2014.10.005 25453829 PMC4254610

[DMM020222C60] Weatherbee S. D., Niswander L. A. and Anderson K. V. (2009). A mouse model for Meckel syndrome reveals Mks1 is required for ciliogenesis and Hedgehog signaling. *Hum. Mol. Genet.* 18, 4565-4575. 10.1093/hmg/ddp422 19776033 PMC2773271

[DMM020222C61] Ye X., Zeng H., Ning G., Reiter J. F. and Liu A. (2014). C2cd3 is critical for centriolar distal appendage assembly and ciliary vesicle docking in mammals. *Proc. Natl. Acad. Sci. USA* 111, 2164-2169. 10.1073/pnas.1318737111 24469809 PMC3926046

[DMM020222C62] Zaghloul N. A. and Brugmann S. A. (2011). The emerging face of primary cilia. *Genesis* 49, 231-246. 10.1002/dvg.20728 21305689 PMC3118297

[DMM020222C63] Zhang Z., Wlodarczyk B. J., Niederreither K., Venugopalan S., Florez S., Finnell R. H. and Amendt B. A. (2011). Fuz regulates craniofacial development through tissue specific responses to signaling factors. *PLoS ONE* 6, e24608 10.1371/journal.pone.0024608 21935430 PMC3173472

