# Supplementary material for: Using the avian mutant talpid2 as a disease model for understanding the oral-facial phenotypes of oral-facial-digital syndrome
Source: Dis Model Mech. 2015 Aug 1;8(8):855–66. doi: 10.1242/dmm.020222 (PMC4527291; doi:10.1242/dmm.020222)
Supplement: Supplementary Material [file supp_8_8_855__index.html]

Supplementary Material 

# Using the avian mutant *talpid**2* as a disease model for understanding the oral-facial phenotypes of Oral-facial-digital syndrome

## DMM020222 Supplementary Material

- Supplementary Material
