# Supplementary material for: Using the avian mutant talpid2 as a disease model for understanding the oral-facial phenotypes of oral-facial-digital syndrome
Source: Dis Model Mech. 2015 Aug 1;8(8):855–66. doi: 10.1242/dmm.020222 (PMC4527291; doi:10.1242/dmm.020222)
Supplement: Supplementary Material [file supp_020222_DMM020222supp.pdf]

Co-IP of C2CD3 and OFD1 - Quantification					
		Co-IP OFD1-myc	IP 3xFLAG-C2CD3	Co-IP/IP	Relative OFD1 Co-IP (to Ctrl)
Trial 1	3xFLAG-$C2CD3^{Ctrl}$	71.60	52.01	1.38	1.00
	3xFLAG-$C2CD3^{fa2}$	27.68	48.00	0.58	0.42
Trial 2	3xFLAG-$C2CD3^{Ctrl}$	72.36	52.67	1.37	1.00
	3xFLAG-$C2CD3^{fa2}$	27.64	47.33	0.58	0.42
Trial 3	3xFLAG-$C2CD3^{Ctrl}$	69.71	56.96	1.22	1.00
	3xFLAG-$C2CD3^{fa2}$	30.30	43.04	0.70	0.58

Fig. S1. Quantification of C2CD3 and OFD1 co-immunoprecipitation.

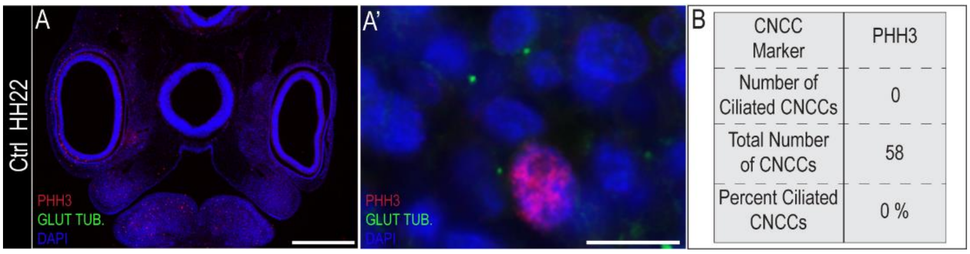

CNCC Marker	PHH3
Number of Ciliated CNCCs	0
Total Number of CNCCs	58
Percent Ciliated CNCCs	0 %

Fig. S2. CNCCs do not extend cilia during mitosis. (A, A') Cranial sections of control HH 22 embryos co-immunostained for glutamylated tubulin (green) and Phospho-histone H3 (PHH3; red). Mitotic cells do not extend a primary cilium. (B) Quantification of *in vivo* primary cilia extension on PHH3-positive CNCCs. Scale bar: (A) 1000 µm, (A') 10 µm.

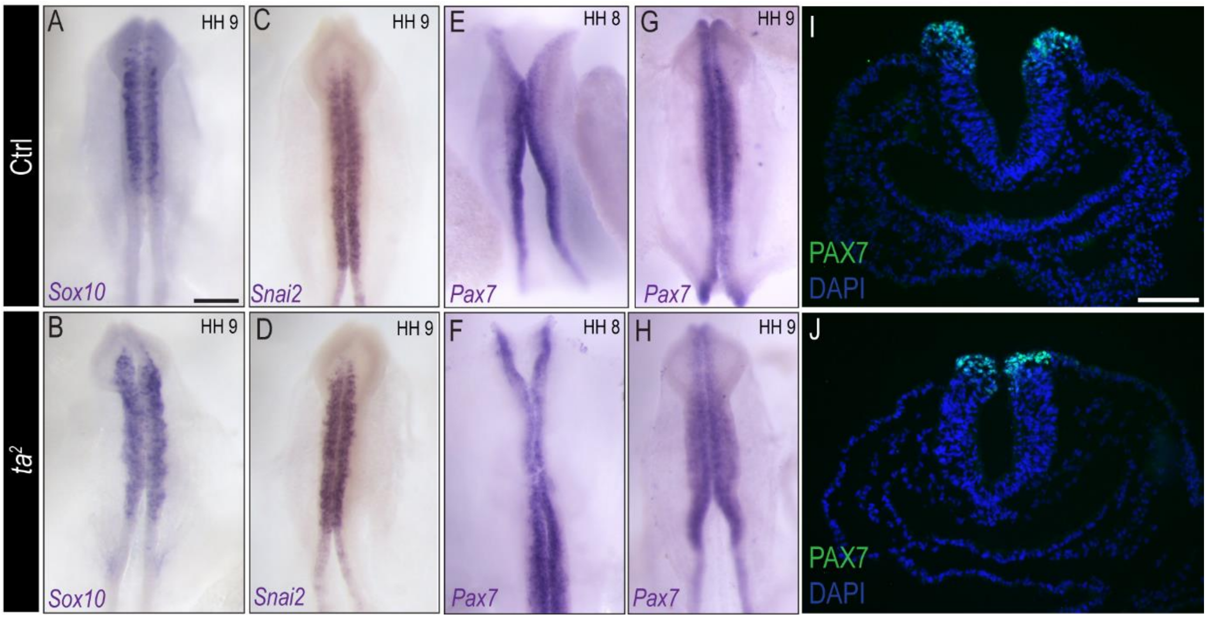

Fig. S3. Loss of C2CD3-dependent ciliogenesis does not affect expression of CNCC specifier genes. (A-H) Whole mount *in situ* hybridization on (A, C, E, G) control and (B, D, F, H) *ta*^{2} embryos at HH 8-9 for (A, B) *Sox10,* (C, D) *Snai2*, and (E-H) *Pax7*. (I, J) Immunostaining for PAX7 (green) on cranial cross sections of control and *ta*^{2} embryos at HH 8$^+$. Scale bars: (A-H) 250 µm; (I-J) 100 µm.

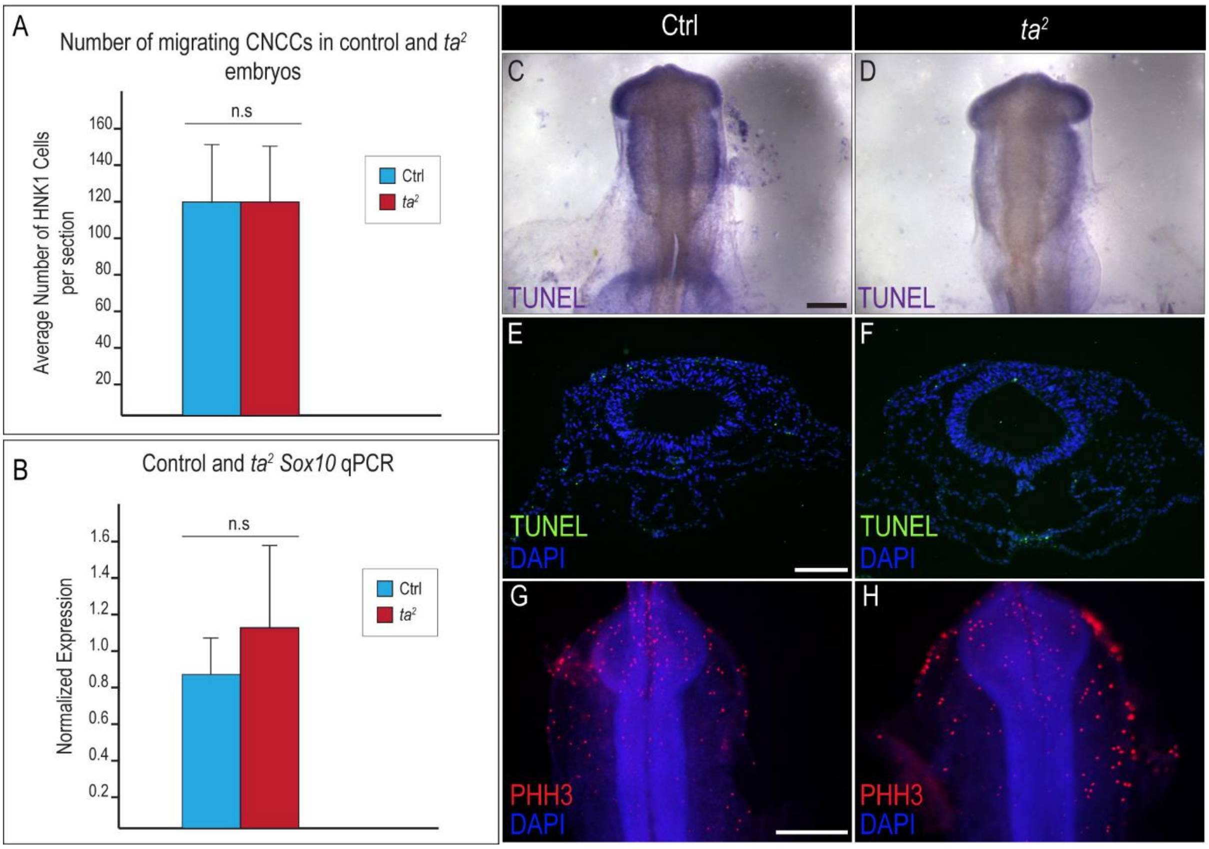

Fig. S4. C2CD3-dependent ciliogenesis does not alter CNCC number, apoptosis or proliferation. (A) Quantitative analysis of the number of HNK1-positive cells/section in control and *ta*^{2} embryos, P=0.99. (B) qRT-PCR analysis for *Sox10* expression in control and *ta*^{2} embryos P=0.17. (C, D) Whole mount and (E, F) sectioned TUNEL staining on HH 10 control and *ta*^{2} embryos. (G, H) Whole mount PHH3 immunostaining (red) on control and *ta*^{2} embryos. Scale bar: (C, D) 400 µm; (E, F) 250 µm; (G, H) 100 µm.

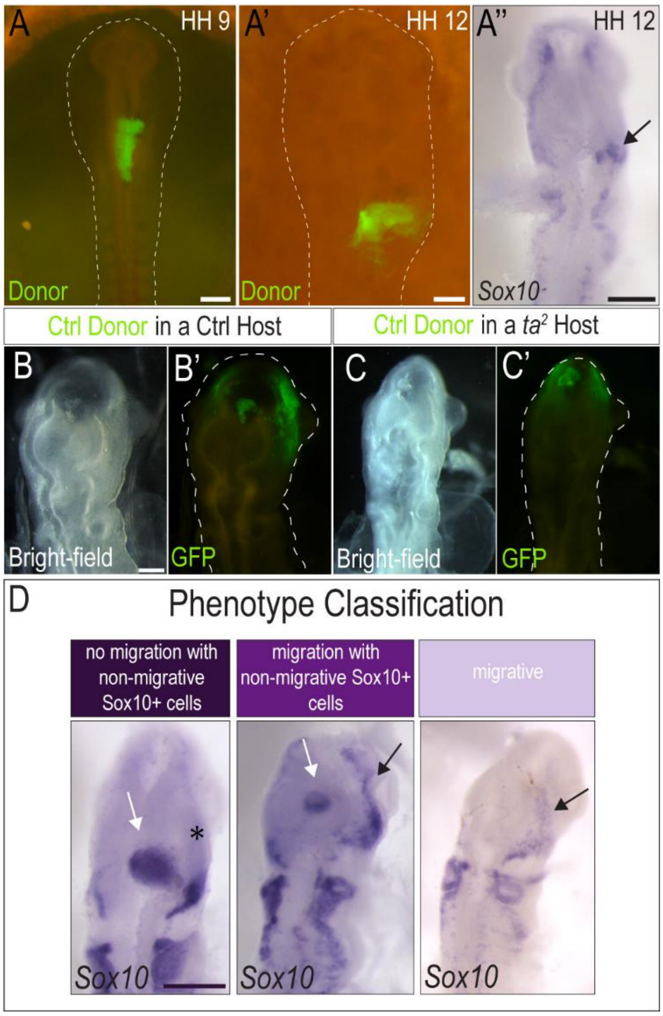

Fig. S5. Dorsal neural tube transplants proof of concept. (A-A'') Sham transplant with a control, GFP-positive donor explant implanted into a control HH 9 host embryo. (A) Dorsal view of sham chimera at the time of transplant. (A') Dorsal view of sham chimera 24 hours after transplant. Donor GFP-positive cells have migrated laterally from the dorsal neural tube. (A'') Dorsal view of *in situ* hybridization for *Sox10* on sham chimera. Donor GFP-positive cells are *Sox10*-positive CNCCs (black arrow). (B-C') Bright-field (B, C) and GFP (B'-C') images for non cell-autonomous transplant experiments from Fig. 5 D', E'. (D) Transplants were categorized in three classes: no migration (black asterisk) with non-migrative *Sox10* positive cells (white arrow); migration (black arrow) with non-migrative *Sox10* positive cells (white arrow); migrative (black arrow). Scale bars: (A) 200 μm; (A') 175 μm; (A'', B-C') 200 μm, (D) 275 μm.

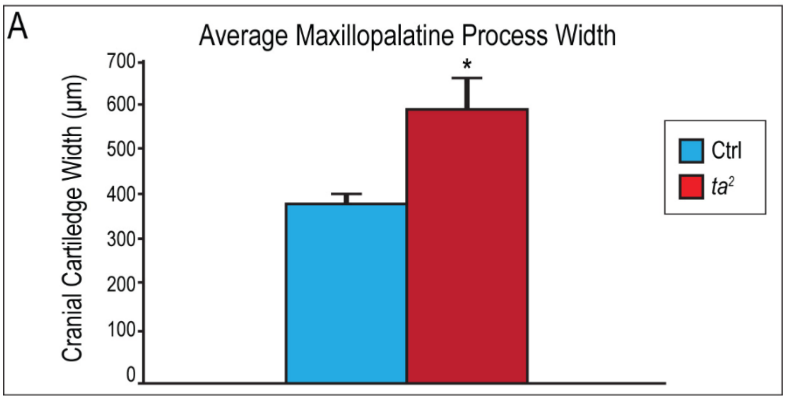

Fig. S6. Alcian blue staining measurements. (A) Quantification of width of the maxillopalatine cartilage, *P<0.05.

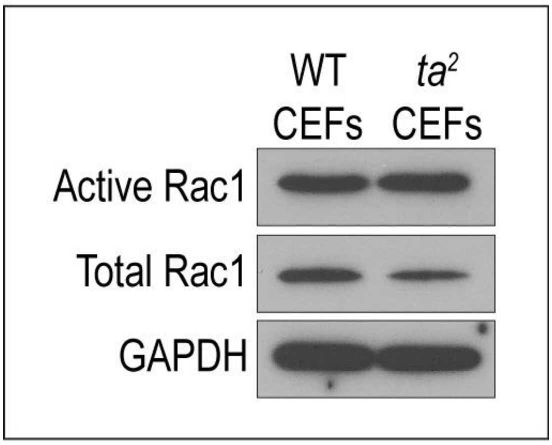

Fig. S7. Rac1 activity is not altered due to loss of C2CD3-dependent ciliogenesis. Western blot for active Rac1, total Rac1, and GAPDH in control and ta^2 CEFs.
